# Nanoscale Mechanism of Moisture-Induced Swelling in Wood Microfibril Bundles

**DOI:** 10.1021/acs.nanolett.2c00822

**Published:** 2022-06-29

**Authors:** Antti Paajanen, Aleksi Zitting, Lauri Rautkari, Jukka A. Ketoja, Paavo A. Penttilä

**Affiliations:** † VTT Technical Research Centre of Finland Ltd, P.O. Box 1000, FI-02044 VTT, Espoo, Finland; ‡ Department of Bioproducts and Biosystems, Aalto University, P.O. Box 16300, FI-00076 Aalto, Espoo, Finland

**Keywords:** Wood-water interactions, Cell wall nanostructure, Cellulose crystallinity, X-ray scattering, Molecular dynamics

## Abstract

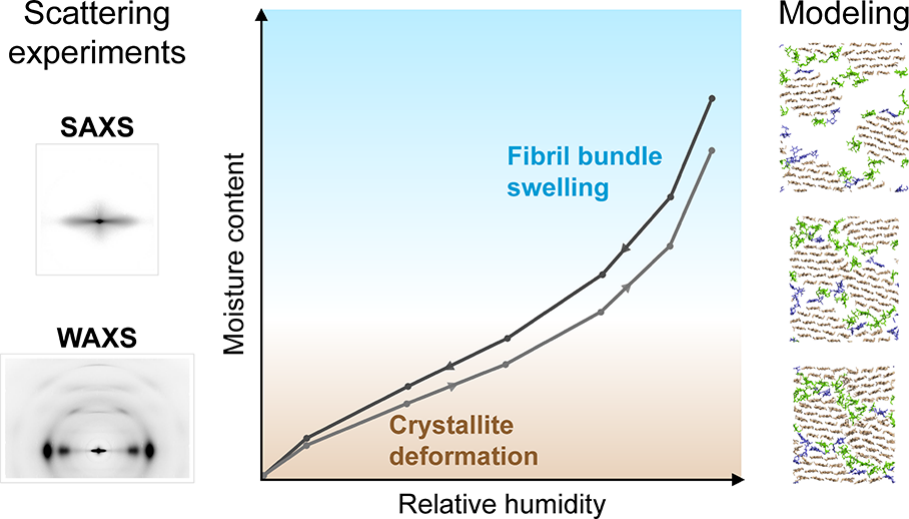

Understanding nanoscale moisture interactions is fundamental to most applications of wood, including cellulosic nanomaterials with tailored properties. By combining X-ray scattering experiments with molecular simulations and taking advantage of computed scattering, we studied the moisture-induced changes in cellulose microfibril bundles of softwood secondary cell walls. Our models reproduced the most important experimentally observed changes in diffraction peak locations and widths and gave new insights into their interpretation. We found that changes in the packing of microfibrils dominate at moisture contents above 10–15%, whereas deformations in cellulose crystallites take place closer to the dry state. Fibrillar aggregation is a significant source of drying-related changes in the interior of the microfibrils. Our results corroborate the fundamental role of nanoscale phenomena in the swelling behavior and properties of wood-based materials and promote their utilization in nanomaterials development. Simulation-assisted scattering analysis proved an efficient tool for advancing the nanoscale characterization of cellulosic materials.

The intriguing characteristics of natural and engineered wood arise from their nanoscale structure, in which water is a key component.^1−5^ Utilization of wood in engineering applications or as a renewable polymer source for advanced materials would benefit from a detailed picture of its structural organization at varied moisture conditions.^6−9^ Furthermore, exploitation of the interactions between cellulose nanostructure and water could enable entirely new nanotechnological applications.^10−12^

The structure of wood is highly hierarchical (Figure 1), consisting of elongated fibrillar structures at all levels. The most important structural level is that of cellulose microfibrils. Their organization directly affects various properties of wood from mechanical behavior to susceptibility to chemical and enzymatic treatments, and eventually to the properties of cellulose-based nanomaterials.^3,13−15^ In the secondary cell walls of wood, microfibrils consisting of parallel cellulose chains are bundled together with hemicelluloses and lignin. The exact molecular-level architecture of the microfibril bundles and the role of water as a structural component are still under active research.^16−18^ A major challenge is the rarity of experimental methods for characterization of the wood nanostructure under various external conditions.^19^

**Figure 1:**
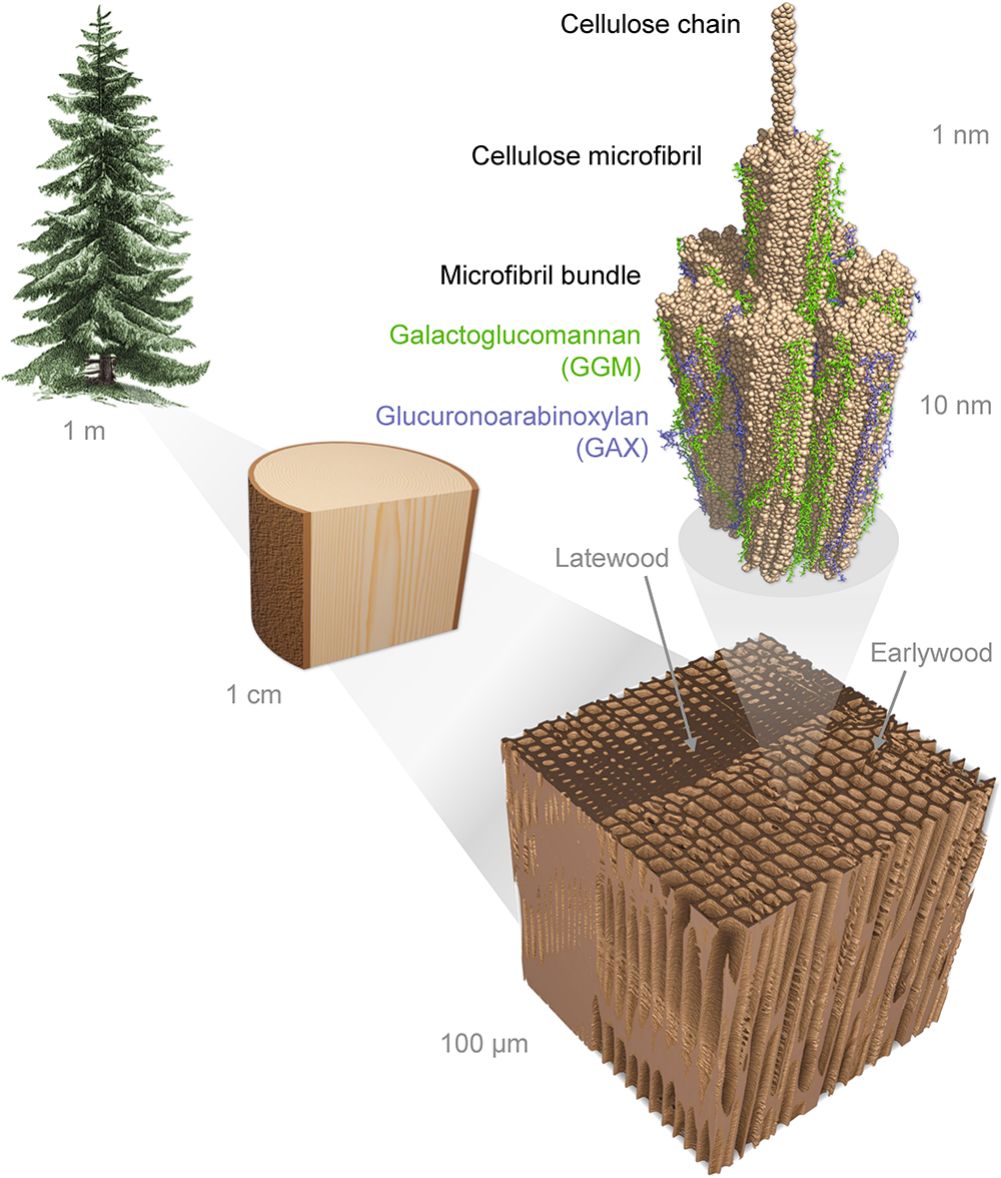
Cartoon representation of the hierarchical structure of spruce wood. The wood tissue (bottom right) consists of elongated tracheid cells, whose secondary cell walls bear the load of the tree. Cellulose microfibril bundles (top right) that consist of microfibrils with a diameter of 2–3 nm are a central component of the secondary cell wall structure. Lignin domains between microfibril bundles are not depicted.

X-ray and neutron scattering enable real-time and non-invasive characterization of wood across multiple length scales and under varying conditions. Results obtained with small-angle X-ray and neutron scattering (SAXS, SANS) show that the distance between microfibrils increases and the microfibril bundles swell with increasing moisture content and vice versa while drying.^20−23^ Wide-angle X-ray scattering (WAXS) and X-ray diffraction studies of wood and other cellulosic materials have shown changes in the positions and widths of the diffraction peaks from crystalline cellulose, although the exact mechanism behind the observed changes is still unclear.^24−33^

A more detailed picture of molecular level phenomena can be obtained by computer simulations.^34^ Atomistic simulations can help explain and visualize scattering results and provide information on the molecular organization within the microfibril bundles. Simulation studies addressing plant cell wall nanostructure have identified different stages in the moisture-induced swelling of wood cell walls, which agree for instance with sorption data.^35,36^ However, a direct link to experimentally observed nanostructural changes in real wood samples has been missing.

The present work combines in situ scattering experiments with atomistic simulations and takes advantage of scattering intensities computed from the simulated structures. This approach allows direct comparison between the modeling and experimental results, and the models visualize the effects detected in scattering experiments.^37^ We have now been able to relate changes in the molecular and microfibril structures to moisture changes in wood cell walls. Our results provide a more concrete and cohesive picture of the nanoscale moisture behavior of wood, which is crucial to understand its properties and processability. It thus enables a more developed utilization of wood for nanotechnological and other applications.

We conducted SAXS/WAXS experiments on never-dried spruce wood (*Picea abies*) in controlled moisture conditions (Figure 2a–c).^38^ The measurement was done first in fully wet state and subsequently at relative humidity (RH) levels from 95% to 10% and back (Figure S1 in Supporting Information (SI)). The strong orientation of wood nanostructure results in anisotropic X-ray scattering patterns (insets in Figure 2a), which allows separation of diffraction peaks from different directions in the crystal lattice. The isotropic scattering component can be subtracted (Figure S2) to obtain specific structural information perpendicular (equatorial) or parallel (meridional) to the longitudinal axis of the microfibrils. The moisture content (MC), defined as the mass ratio of water and the dry sample, at different RHs was determined by separate gravimetric measurements, and the value 41% for water-saturated cell walls was taken from literature.^39^ The sample contained 44% cellulose, 17% galactoglucomannan (GGM), 7% glucuronoarabinoxylan (GAX), and 27% lignin, as measured by dry weight (Table S1), and 43% latewood by cell wall volume, determined with X-ray microtomography (Figure S5).

**Figure 2:**
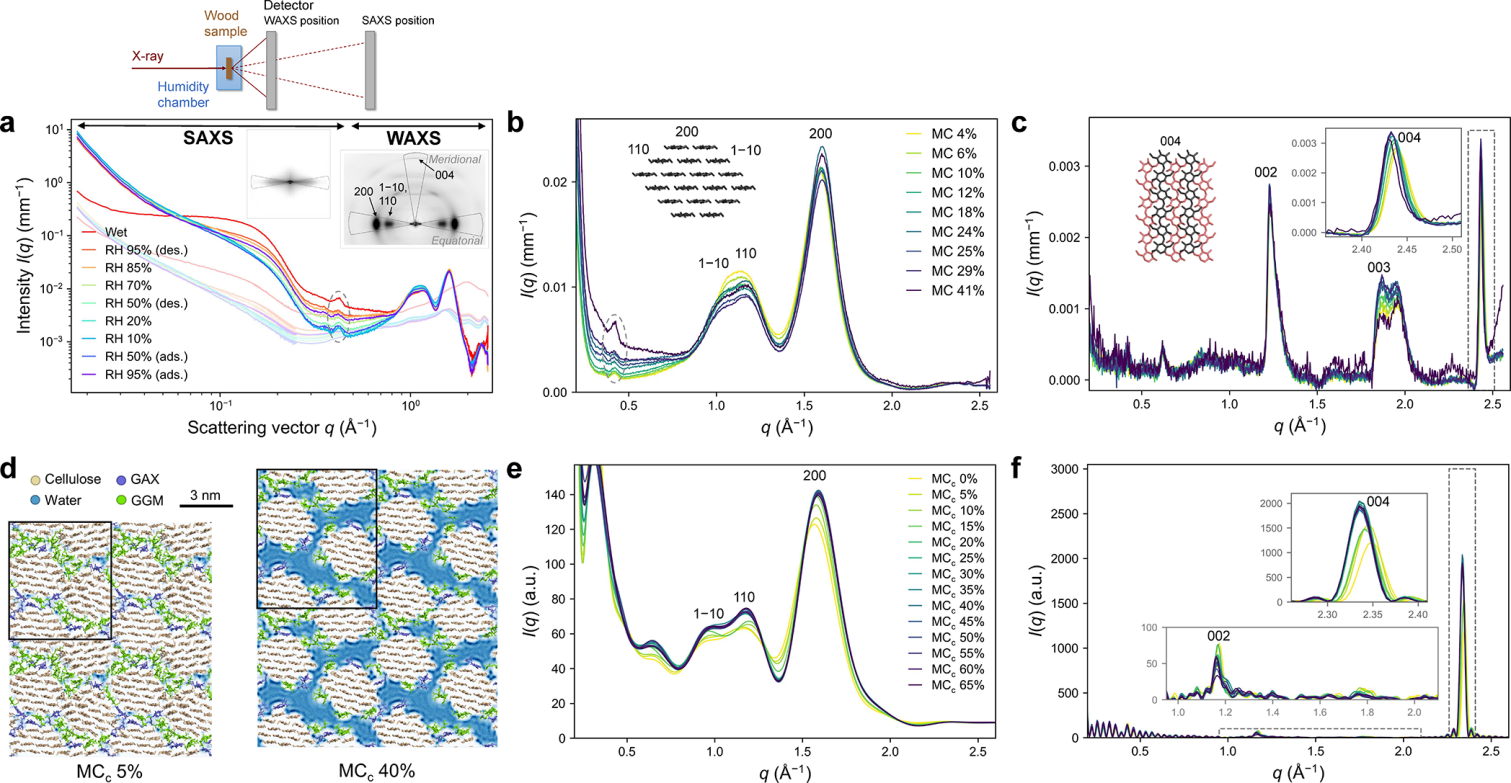
X-ray scattering at different moisture conditions, as observed experimentally (a–c) and computed (e,f) from molecular models such as in (d). (a) Equatorial anisotropic SAXS and WAXS intensities on double-logarithmic axes with the isotropic scattering contribution shown with light-colored lines. A schematic representation of the experiment is shown above the image. The main reflections from cellulose I_β_ and the equatorial and meridional integration sectors are indicated in the two-dimensional scattering patterns shown as insets. (b) Equatorial and (c) meridional anisotropic WAXS intensities, labeled with the corresponding moisture content (MC). The peak marked by a dashed line in (a) and (b) is due to unsuccessful background subtraction (Kapton windows), see SI for details. (d) Molecular model of a microfibril bundle of four microfibrils in a periodic simulation domain (domain outlined in black). MC_c_ refers to the moisture content relative to the carbohydrates. (e) Equatorial and (f) meridional scattering intensities computed fibril-by-fibril from the model shown in (d), excluding water, labeled with the MC_c_ (see Figure S7 for scattering intensities computed from all models).

To relate our experimental observations to real-space molecular configurations, we conducted molecular dynamics (MD) simulations of microfibril bundles under various moisture conditions (see Figure S3). A periodic system of four microfibrils in a hexagonal arrangement (Figure 2d) was simulated with precise control of MC, and a non-periodic bundle of seven microfibrils was used complementarily. In addition, results from a periodic model of one microfibril are presented in the SI (see Figure S3 for all models). The models were built of cellulose microfibrils consisting of 18 cellulose chains^40^ initially in the cellulose I_β_ crystal lattice,^41^ with 2-3-4-4-3-2 parallel chains in each hydrogen-bonded layer^42^ and no extensive disordered regions along the fibril length.^43^ In the non-periodic models, the fibrils could twist around their long axis, whereas in the periodic models the fibrils were fixed by boundary constraints. The cellulose fibrils were coated by GGM and GAX chains, with their proportions reflecting the chemical composition of the wood sample (Table S1) and following the recently outlined molecular architecture of spruce wood.^17^ Water was able to penetrate the hemicellulose matrix between the microfibrils and to induce swelling of the aggregate (SI video). As a preliminary test of the models’ feasibility, we confirmed that the predicted mass density and microfibril packing distance at different MCs were consistent with typical values of the cell wall density (Figure S6) and small-angle scattering results from the literature.^20−23,44^

To enable direct comparison between the simulation and scattering results and to gain insight into their interpretation, we computed scattering intensities based on the atomistic models (Figure 2e,f). The calculation was performed microfibril-by-microfibril, that is, by separating individual microfibrils from the bundles and treating them as single scatterers, assuming random orientation around the fibril axis.^45^ This yielded scattering intensities comparable to the equatorial and meridional anisotropic intensities from the SAXS/WAXS experiments. To study the contribution of hemicelluloses to the scattering, the intensities were also computed without the hemicelluloses. Scattering from a complete non-periodic bundle of seven fibrils was computed to illustrate the effect of microfibril aggregation. The WAXS intensities exhibited the cellulose I_β_ diffraction pattern, whereas the artificial lack of cell wall material around the fibrils introduced strong oscillations to the intensity in the SAXS range, that is, at values of the scattering vector *q* below 0.8 Å^–1^.

Perhaps the simplest fundamental information available from WAXS data of crystalline cellulose are the lattice spacings (*d*_*hkl*_), which are related to the locations of the diffraction peaks (with Miller indices *hkl*) in the reciprocal space (described by scattering vector **q**). The anisotropic WAXS intensities showed clear changes in the positions of the diffraction peaks (Figures 2b,c, S8). As the sample dried, the shifts of the most intense diffraction peaks (*hkl* = 200 and 004) indicated an increase in the lattice spacing perpendicular to the hydrogen-bonded layers (*d*_200_) and a decrease along the fibril axis (*d*_004_) and partial recovery when increasing the RH again (Figure S8c). The incomplete recovery with respect to RH is related to the commonly known sorption hysteresis of wood (difference between adsorption and desorption isotherms).^2^ When examined against the actual MC of the samples, a reversible trend is revealed (Figure 3a). The experimental results agreed well with lattice spacings determined directly from the models (Figure 3a). In addition, the models showed that the variation of *d*_200_ and *d*_004_ was larger close to the microfibril surfaces and increased at lower MCs (Figure S9). Only the absolute level of *d*_004_ deviated from the experiments, which is characteristic of the force field used in the simulations (GLYCAM06)^46^ and also attributable to the natural variation of *d*_004_, possibly due to residual growth stresses.^18^ When fitting the computed intensities in the same way as the experimental WAXS data (Figures 3a, S10), the expected behavior was found for *d*_200_ and *d*_004_.

**Figure 3:**
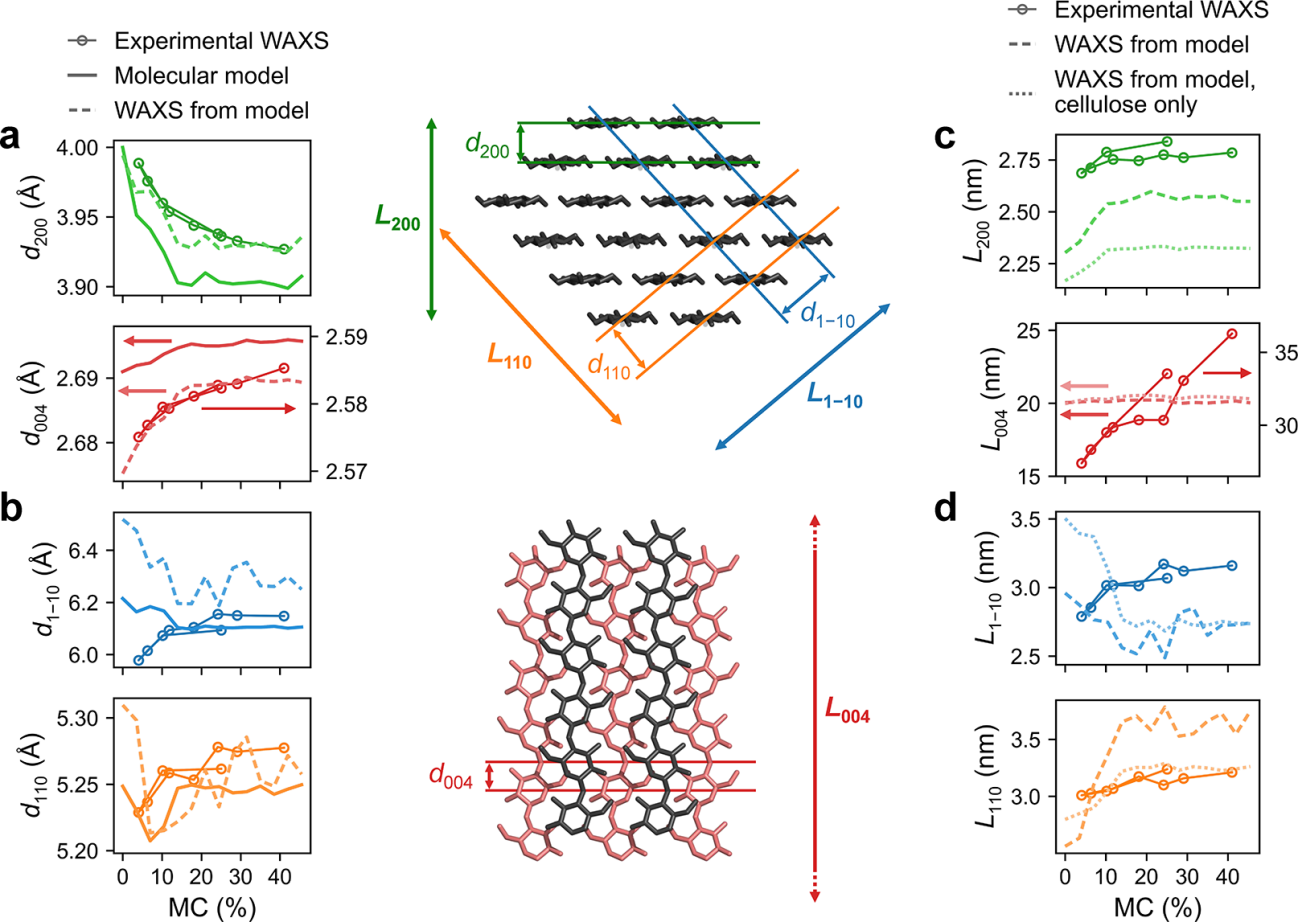
Moisture-dependence of the crystalline parameters based on WAXS experiments and molecular models. (a,b) Lattice spacings *d*_*hkl*_ and (c,d) crystal size *L*_*hkl*_ corresponding to different directions *hkl* in the crystal, as determined experimentally and from the periodic bundle model either directly (see Figure S4) or from computed scattering intensities. The MC for the models (MC_c_) has been multiplied by 0.7 (approximate mass ratio of carbohydrates and total dry mass) to make it comparable with results from complete cell walls. The experimental values shown for the 200 peak correspond to a weighted average of a bimodal size distribution of crystallites (see SI for details). Note the separate vertical axes for experimental (right) and model-based (left) results for *hkl* = 004 in (a,c). See Figures S9, S10, and S11 for more results.

Changes in the diagonal directions of the microfibril cross-section (*d*_11̅0_ and *d*_110_) were less systematic (Figure 3b). The experiments indicated a decrease in both with drying, whereas *d*_11̅0_ in the models showed a change in the opposite direction and *d*_110_ remained rather constant. In the models, these lattice spacings were sensitive to the specific aggregation of the microfibrils, and similar to *d*_200_ and *d*_004_ their variations were strongest on the microfibril surfaces and increased towards the dry state (Figure S9). In the computed scattering intensities, fits to the 11̅0 and 110 peaks often reflected the changes in lattice spacings determined directly from the models (Figures 3b, S10, and S11a). When examining the results as lattice parameters of the monoclinic unit cell, the largest discrepancies between the experimental and model-based results were found in the lattice constant *b* (in the hydrogen-bonded layer, perpendicular to the chain axis) and the monoclinic angle γ (Figure S11b). These observations indicate that the 11̅0 and 110 diffraction peaks are sensitive to the molecular configurations at microfibril surfaces and fibrillar aggregation,^47^ which thus contribute to the WAXS analysis of cellulosic samples.

In the experimental WAXS data (Figures 2b,c), the diffraction peaks broadened with drying. Such broadening can originate from increased variation of the lattice spacings or a decrease in the coherence length of the crystalline order (crystal size). As both interpretations relate broader peaks with less ordered structure, we discuss the peak broadening using the term “crystal size”, *L*_*hkl*_, which is inversely proportional to the peak width after correcting for instrument-related broadening (SI eq S2). The parameter *L*_*hkl*_ decreased with drying for all peaks in the experimental WAXS data and for most peaks in the intensities computed from most models (Figures 3c,d, S10). The 11̅0 peak in the computed scattering intensities showed an opposite trend, which could be an effect of the SAXS-range oscillations disturbing the fitting. By comparing the crystal size *L*_200_ computed from the same model either including or excluding the scattering from hemicelluloses (Figure 3c), a contribution of 0.2 nm to the crystal size can be attributed to the hemicelluloses. The peak broadening and crystal size are therefore sensitive to changes in the organization of hemicelluloses on the microfibril surfaces, which varied with MC in the molecular models. However, the observed broadening of the modeling-based lattice spacing distributions with drying (Figure S9) suggests that the experimental WAXS peak broadening could be largely due to a drying-induced distortion of the crystal structure.

Various explanations have been suggested for the shifts of diffraction peaks with moisture,^26,27,30−33,48^ and their compatibility with our modeling results is discussed in Table S2. Our conclusion is that although the drying of the matrix polymers (hemicelluloses) may introduce compressive forces especially along the fibril,^24^ the most significant deformations of the crystallites at low MCs should originate from an overall reorganization of the interfibrillar matrix and fibrillar aggregation. This explanation is supported by visual inspection of the models, where the surfaces of the microfibrils become especially distorted close to the dry state (see, e.g., Figure 4e). It may bear some similarity to grain boundary effects in polycrystalline solids.^49^ The general observation of drying-induced disorder in cellulose crystallites is in line with the results of several other studies utilizing different methods.^29,50−52^

**Figure 4:**
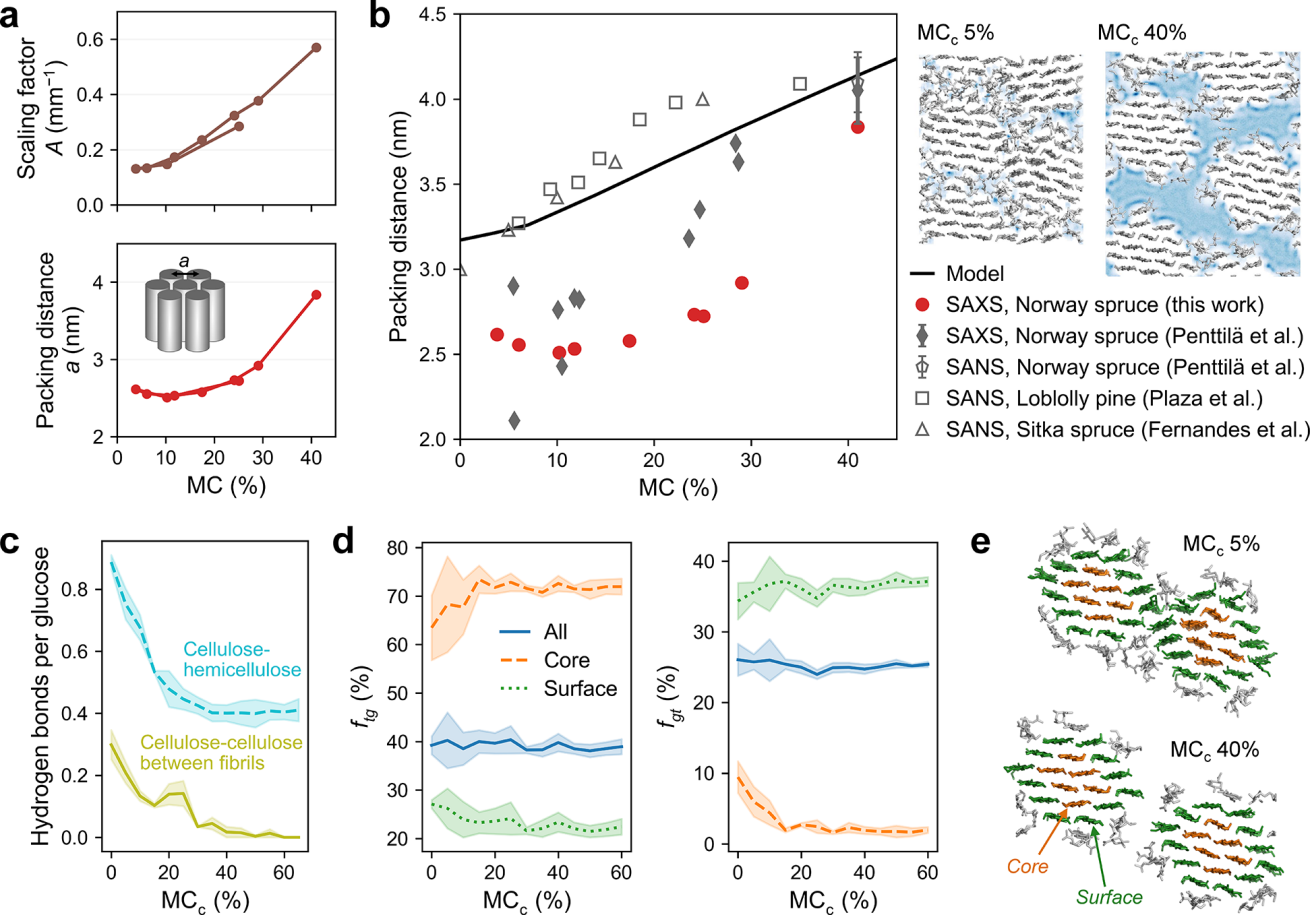
Moisture-related swelling of microfibril bundles based on scattering analysis and modeling. (a) Results of SAXS fits using the WoodSAS model (SI eq S3)^22^ (see Figure S13 for all results). (b) Microfibril packing distance as a function of MC (0.7 × MC_c_) as determined from the periodic bundle model (see Figure S15 for non-periodic model) and a comparison to experimental SAXS and SANS results^20,22,23,29,58,59^ (MC of 41% used for Norway spruce in water-saturated state;^39^ error bars correspond to standard deviation between samples). Snapshots of the periodic model with density of water in blue are shown on the right. (c) Number of hydrogen bonds as a function of MC_c_ (see Figure S16 for all results). (d) Fractions (*f* in %) of primary alcohol group conformers (*tg*, *trans–gauche*; *gt*, *gauchetrans*) as a function of MC_c_, shown separately for all cellulose chains and chains at microfibril core and surface (see Figure S17 for all results). In panels c and d, the lines correspond to the mean of all four fibrils and the filled areas to ±1 standard deviation of the means of each fibril. (e) Simulation snapshots of two microfibrils of the periodic model (cellulose in green and orange, hemicelluloses in gray), which illustrates the deformation of cellulose crystals in the dry state. This partially explains the changes in lattice spacings and experimentally determined crystal size (Figure 3).

Computed scattering intensities from the whole bundle of seven microfibrils (Figure S7d) gave additional insight into the effects of fibrillar aggregation on the WAXS data. The equatorial diffraction peaks were sensitive to the orientation of the microfibrils around their long axis, which occasionally allowed co-crystallization of neighboring fibrils with matching orientations. In particular, the 200 diffraction peak of the non-periodic bundle showed a broader and narrower contribution (Figure S12c,d). This was used as a basis for fitting the WAXS data of the 200 peak with two overlapping peaks, putatively originating from single and stacked microfibrils. Our models also exhibited limitations in the meridional scattering. The computed scattering intensities (Figures 2f, S7) successfully reproduced the 002 and 004 peaks originating from cellulose but not the contribution around the “forbidden” 003 reflection.^53^ A moisture-sensitive 003 peak has been observed in experiments (e.g. Figure 2c, *q* = 1.9 Å^–1^) and previously assigned to the organization of hemicelluloses.^18^ As this peak was absent in all computed scattering regardless of the inclusion of hemicelluloses, it probably originates from structures that were not well presented by our models. Thus, both the aggregation of the microfibrils and the configuration of hemicelluloses on the microfibril surfaces likely affect the results of WAXS analysis of real samples, and scattering intensities computed from molecular models can help resolve these contributions. We did not notice any major effects due to microfibril twisting^54,55^ when comparing the computed scattering intensities and moisture behavior of the non-periodic (twisting) and periodic (non-twisting) fibril models.

The equatorial anisotropic SAXS data (Figures 2a, S13) contains information on the lateral dimensions and packing of the microfibrils. They can be analyzed by a small-angle scattering model tailored for wood (WoodSAS model, eq S3),^22^ which approximates the microfibrils as hexagonally packed cylinders. This fitting reproduced the previously reported, reversible trends:^29^ a decrease in the microfibril packing distance and the cylinder scaling factor (sensitive to scattering contrast) with drying, as water withdraws from the space between the microfibrils (Figure 4a). This (de)swelling of the microfibril bundles is responsible for a large part of the dimensional changes of the cell wall, and therefore of the entire macroscopic sample.^23^ At the macroscopic scale, the sample width decreased by 7% between MCs 29% and 4% (Figure S14), which is a slightly smaller change than the shrinkage of 10% in the microfibril bundles detected by SAXS over the same MC range. This difference is explained by other cell wall layers and different parts of the wood tissue restraining the macroscale swelling behavior.^36^

The molecular models successfully reproduced the experimentally observed linear swelling of the microfibril bundles above a few per cent MC with values of the fibril packing distance comparable to those from scattering experiments (Figures 4b). The bundle swelling in the models was accompanied by a decrease in hydrogen bonds between hemicelluloses and cellulose and between surface chains of neighboring fibrils, which are in direct contact at low MCs (Figures 4c, S16). This illustrates a close association of the hemicelluloses on the microfibril surfaces at low MCs and demonstrates the essential role of the hemicellulose matrix in absorbing water and inducing swelling of the microfibril bundles. In the computed scattering intensities, we detected a moisture-sensitive scattering contribution at the crossover region between SAXS and WAXS (*q* = 0.4–0.8 Å^–1^, Figures 2e, S7), which is visible also in experimental data (Figure 2a,b). We tentatively assign this intensity increase to the hydration and reorganization of the hemicellulose shell around the microfibrils. Another indication of a reorganization of the disordered polymer matrix is the shift of the isotropic WAXS intensity maximum to lower *q* with drying (Figure 2a).^24^

The swelling of the microfibril bundles and reorganization of the matrix polymers have implications for the mobility of water within the bundles. As reported in a related study,^44^ the diffusivity of interfibrillar water decreases rapidly with decreasing MC. This can be assigned to a reduced ability of sparsely distributed water molecules to proceed in an amorphous polysaccharide matrix.^56^ The current models reproduce the exponential decrease of the diffusion coefficient of bound water as observed in ^1^H-NMR experiments of cellulosic fibers,^57^ which provides further support for the used model geometry.

Throughout the X-ray scattering and modeling results, we noticed that the changes in the SAXS and WAXS regimes, that is, at different hierarchical levels, dominated at different MC ranges. The microfibril packing distance changed mostly above MC 15% (Figure 4a), accompanied by slight changes in the microfibril orientation (Figure S18), and remained constant below it. On the contrary, the lattice spacings and the crystal size showed only small changes at high MCs and changed most significantly and with largest variations at MCs below 15% (Figure 3). A closer look into the models revealed a clear deformation of the crystals at low MCs. This manifested, among others, as an abrupt transition in the primary alcohol group conformations of the core chains from *tg* (crystalline cellulose I_β_) to *gt* with decreasing MC (Figure 4d). Simultaneously, the fraction of *tg* conformers increased in the surface chains, as they occasionally adapted to the crystal structure of the neighboring fibrils (Figure 4d,e). Other works have reported abrupt changes in the mechanical properties of wood, its susceptibility to fungal degradation, and water diffusion and hydrogen bonding at similar MCs,^60−62^ which coincides with the softening of hemicelluloses.^3^ A previous diffraction study also found the largest moisture-related changes in the lattice parameters of spruce wood cellulose below MC 13%.^33^ Nevertheless, the detailed mechanisms and interactions behind these changes and the exact role of water in this structural transformation has so far remained elusive.

The results of our combined X-ray scattering experiments and MD simulations, supported by previous findings,^2,3,20,23,33,35,36^ can be taken together to outline a coherent picture of the structural changes in microfibril bundles at different MC ranges (Figure 5). Even though individual pieces of this picture existed before, our current results, obtained by studying the same system with simulations and scattering experiments, helped to link the different aspects together to form a more complete image of this complex phenomenon. The exact mechanisms controlling the behavior, such as the transfer from one stage to the other, remain intriguing subjects for further studies for which the methodology presented here provides excellent tools. On the basis of our observations, the swelling of microfibril bundles originates from the sorption of water to the hemicelluloses and microfibril surfaces. However, fibrillar aggregation also plays an essential role in the moisture behavior, introducing deformations to the cellulose crystals closer to the dry state. Considering the inherent hierarchical structure formed by fibrillar units is therefore indispensable for understanding the structural and functional properties of materials based on plant cell walls. The new information provides means to understand and control the fundamentally important swelling of wood in the nanoscale, which enables the design of novel wood-based nanomaterials and other applications.

**Figure 5:**
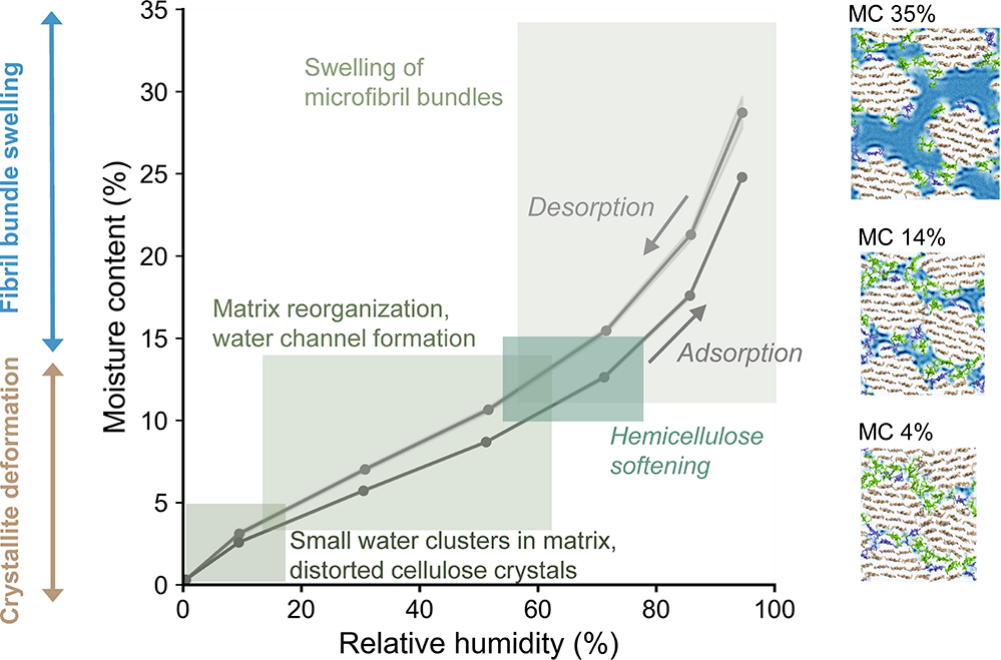
Summary of structural changes taking place in microfibril bundles in spruce wood cell walls at different MC ranges, illustrated in connection with a sorption isotherm (circles indicating the data points). In the dry state (MC close to 0%), the cellulose microfibrils are tightly packed and locked into a position where the cellulose crystallites are deformed due to interaction with the matrix polymers and neighboring fibril surfaces. Once some water becomes available (MC a few per cent), it penetrates the matrix and forms small clusters. Further increase of the MC allows the interfibrillar matrix to reorganize and to accommodate more water, which releases the deforming stresses on the microfibrils and allows them to adopt a higher level of crystalline order. At around MC 10–15%, the individual water clusters have grown and merged enough to occupy the spaces between the microfibrils, and the matrix softens in a process reminiscent of a glass transition. Above this point, the matrix can deform more easily, and it expands to accommodate more water in continuous channels, and the fibril bundles swell causing an increase in the microfibril packing distance and allowing faster diffusion of water. Simulation snapshots with MC referring to 0.7 × MC_c_ are shown on the right (see Figure S19 for all).

Our study shows clear methodological advances when combining molecular simulations and X-ray scattering for a more accurate interpretation of nanoscale interactions between water and the main structural components of plant cell walls. Our up-to-date model of microfibril bundles allowed transferring from a conceptual picture to a quantitative description of the moisture behavior of wood cell walls. It highlights the role of the hierarchical structure of the bundles, in which their deswelling eventually turns into a deformation of the cellulose crystals with continued drying. This coherent understanding forms a basis for quantitative prediction and modeling of cellulosic materials. The approach is not limited to moisture behavior of wood but can be adapted to other cellulosic nanomaterials and for studies including mechanical stresses, mechanosorption, sorption hysteresis, and irreversible effects of drying.

## Supporting Information Available

Full description on materials and methods including sample preparation, chemical analysis, X-ray scattering, X-ray microtomography, sorption experiments, and modeling; supplementary results including more detailed scattering, modeling, tomography and chemical analysis results, and a table summarizing the suggested mechanisms for cellulose lattice deformations with moisture changes (PDF)
Video of a swelling microfibril bundle based on the simulations of the periodic model with four fibrils (AVI)

## Supplementary Material

nl2c00822_si_001.pdf

nl2c00822_si_002.avi

## ABBREVIATIONS

GAX: glucuronoarabinoxylan
GGM: galactoglucomannan
MC: moisture content
MC_c_: moisture content relative to carbohydrates
MD: molecular dynamics
RH: relative humidity
SANS: small-angle neutron scattering
SAXS: small-angle X-ray scattering
WAXS: wide-angle X-ray scattering.
