# Supplementary material for: Nanoscale Mechanism of Moisture-Induced Swelling in Wood Microfibril Bundles
Source: Nano Lett. 2022 Jun 29;22(13):5143–50. doi: 10.1021/acs.nanolett.2c00822 (PMC9284609; doi:10.1021/acs.nanolett.2c00822)
Supplement: Supplementary file 1 — nl2c00822_si_001.pdf [file nl2c00822_si_001.pdf]

Supporting Information

Nanoscale Mechanism of Moisture-Induced Swelling in Wood Microfibril Bundles

Antti Paajanen[1,], Aleksi Zitting[2], Lauri Rautkari[2], Jukka A. Ketoja[1], Paavo A. Penttilä[2,]**

1. VTT Technical Research Centre of Finland Ltd,

P.O. Box 1000, FI-02044 VTT, Espoo, Finland

2. Department of Bioproducts and Biosystems, Aalto University,

P.O. Box 16300, FI-00076 Aalto, Espoo, Finland

* Corresponding authors

Contents

Page

1. Materials and methods

1.1 Wood samples S2

1.2 Compositional analysis S2

1.3 X-ray scattering; Figures S1, S2 S2

1.4 Dynamic vapor sorption S6

1.5 X-ray microtomography S7

1.6 Molecular simulations; Figures S3, S4 S7

1.7 Computed scattering intensities and their analysis S10

2. Supplementary results

Tables S1, S2 S12

Figures S5–S19 S14

References S27

1. Materials and methods

1.1 Wood samples

Stem discs from approximately 1 m height in a freshly felled mature Norway spruce (*Picea abies*) tree grown in Southern Finland were sealed into plastic bags and stored in a freezer at –20°C. Wood blocks with tangential width of approximately 10 mm were separated from the sapwood portion between annual rings 34 and 52 using a handsaw. Samples for the compositional analysis and sorption isotherm measurement were ground in a Wiley mill. For all other experiments, the blocks were further cut into 1-mm thick tangential-longitudinal sections using a sliding microtome and stored in deionized water at 4°C. Finally, samples with approximate dimensions of 1×1×8 mm^3 (radial×tangential×longitudinal) were cut with a razor blade from a section containing both earlywood and latewood.

1.2 Compositional analysis

Wood samples ground in a Wiley mill were air dried and further ground using Fritch Pulverizette 14 mill. The extractives were determined gravimetrically after Soxhlet extraction with heptane. To determine the carbohydrate and lignin composition, the samples were hydrolyzed first with 72% (w/w) sulfuric acid for 60 min at 30°C and then autoclaved with 4% (w/w) sulfuric acid for 60 min. The resulting monosaccharides were determined by high-performance anion-exchange chromatography with pulsed amperometric detection (HPAEC-PAD, Dionex ICS 5000 equipped with CarboPac PA20 column) according to NREL method.[1,2] The polysaccharide content in the samples was calculated from the corresponding monosaccharides using an anhydro correction of 0.88 for pentoses and 0.9 for hexoses. Klason lignin content, i.e., the insoluble residue from the hydrolysis, was determined gravimetrically. Acid soluble lignin in the hydrolysate was detected at wavelengths 215 nm and 280 nm using the equation described by Goldschmid.[3] The sample composition, i.e., mass fractions of cellulose, galactoglucomannan (GGM), glucuronoarabinoxylan (GAX), and lignin, was calculated assuming compositional ratios of 1:4:1 for glucan, mannan and galactan in GGM and 12:2:1 for xylan, glucan and arabinan in GAX.

1.3 X-ray scattering

Experiments. For a measurement in the fully wet state, a wood sample was taken from deionized water, wrapped in Mylar foil and inserted in a humidity-controlled sample stage (Xenocs) adjusted to relative humidity (RH) 95% and temperature 25°C. The sample was oriented with its tangential-longitudinal face perpendicular to the incoming X-ray beam and fiber axis vertical. Small and wide-angle X-ray scattering (SAXS and WAXS) were measured with a Xenocs Xeuss 3.0 C SAXS/WAXS device, equipped with a GeniX 3D Cu source (wavelength λ = 1.542 Å) and EIGER2 R 1M detector. WAXS data at sample-to-detector distance 153 mm was collected by utilizing the virtual detector and line eraser modes, with 6×2 images (exposure time

400–500 s each) stitched together to cover a gapless q-range of 0.04–2.6 Å^{-1} (scattering vector $q = 4\pi \sin(\theta)/\lambda$ with scattering angle 2θ). SAXS data at sample-to-detector distance 414 mm was collected using the line eraser function, in which two images (exposure time 400–600 s each) were taken to cover a gapless q-range of 0.017–0.32 Å^{-1}. After the measurement in fully wet state, the Mylar foil around the sample was removed, and the sample was allowed to equilibrate at RH 95% for 10 h prior to data collection. The RH was varied stepwise from 95% to 85%, 70%, 50%, 20%, 10%, 50% and back to 95%, with collection of SAXS/WAXS data after an equilibration time of 2.5–59 h at every step (see Figure S1 for details). Background scattering corresponding to an empty sample stage (and Mylar foil for the wet sample) was measured separately for every RH step. In addition, the macroscopic dimensional changes of the sample in the tangential direction (perpendicular to the direction of the X-ray beam) were measured by performing a horizontal scan with a high-resolution X-ray beam. The tangential width of the sample was determined as integral breadth of the density profile obtained by the scanning.

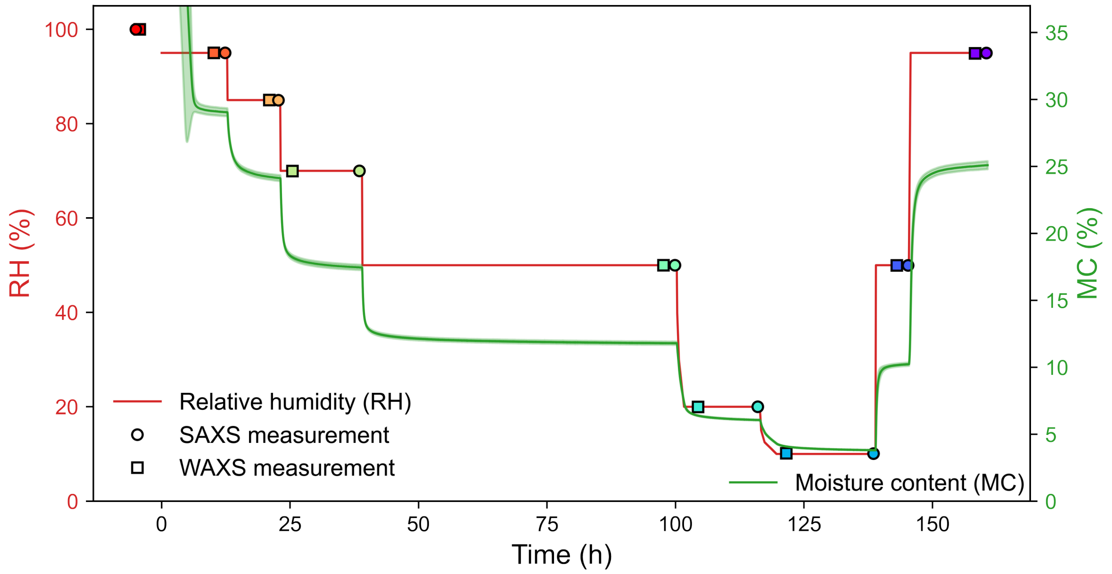

Figure S1. Complete relative humidity (RH; vertical axis on the left) cycle used in the SAXS/WAXS and dynamic vapor sorption (DVS) experiments, with the points of the scattering measurements indicated by symbols, and the associated moisture content (MC; vertical axis on the right) of the sample determined by DVS. The SAXS/WAXS data points next to time 0 h refer to the sample measured in wet state, wrapped in Mylar foil.

Data processing. The intensities in the complete two-dimensional scattering images were corrected for cosmic background and normalized by transmitted beam intensity, after which the scattering from an empty sample holder was subtracted. Due to the strongly anisotropic scattering from the wood sample, subtracting the contribution of Kapton windows on the path of the X-ray beam was not entirely successful, leaving a peak at $q = 0.4$ Å^{-1} (marked in Figure 2a,b in the main article). Normalization to absolute units (mm^{-1}) was done using a glassy carbon standard and an approximate sample thickness of 1 mm. The corrected two-dimensional scattering patterns were further corrected for solid angle and converted to polar presentation (azimuthal angle ϕ vs. q) using the PyFAI Python library.[4] The isotropic scattering component was determined as the azimuthal minimum at each value of q and subtracted from intensities integrated over 25°-wide azimuthal sectors around the equatorial and meridional scattering streaks (see Figure S2 for examples). This yielded the equatorial and meridional anisotropic intensities, respectively. A smoothing operation (to reduce noise) was carried out for the isotropic WAXS intensity prior to subtraction. The equatorial anisotropic intensities from the WAXS and SAXS ranges were merged.

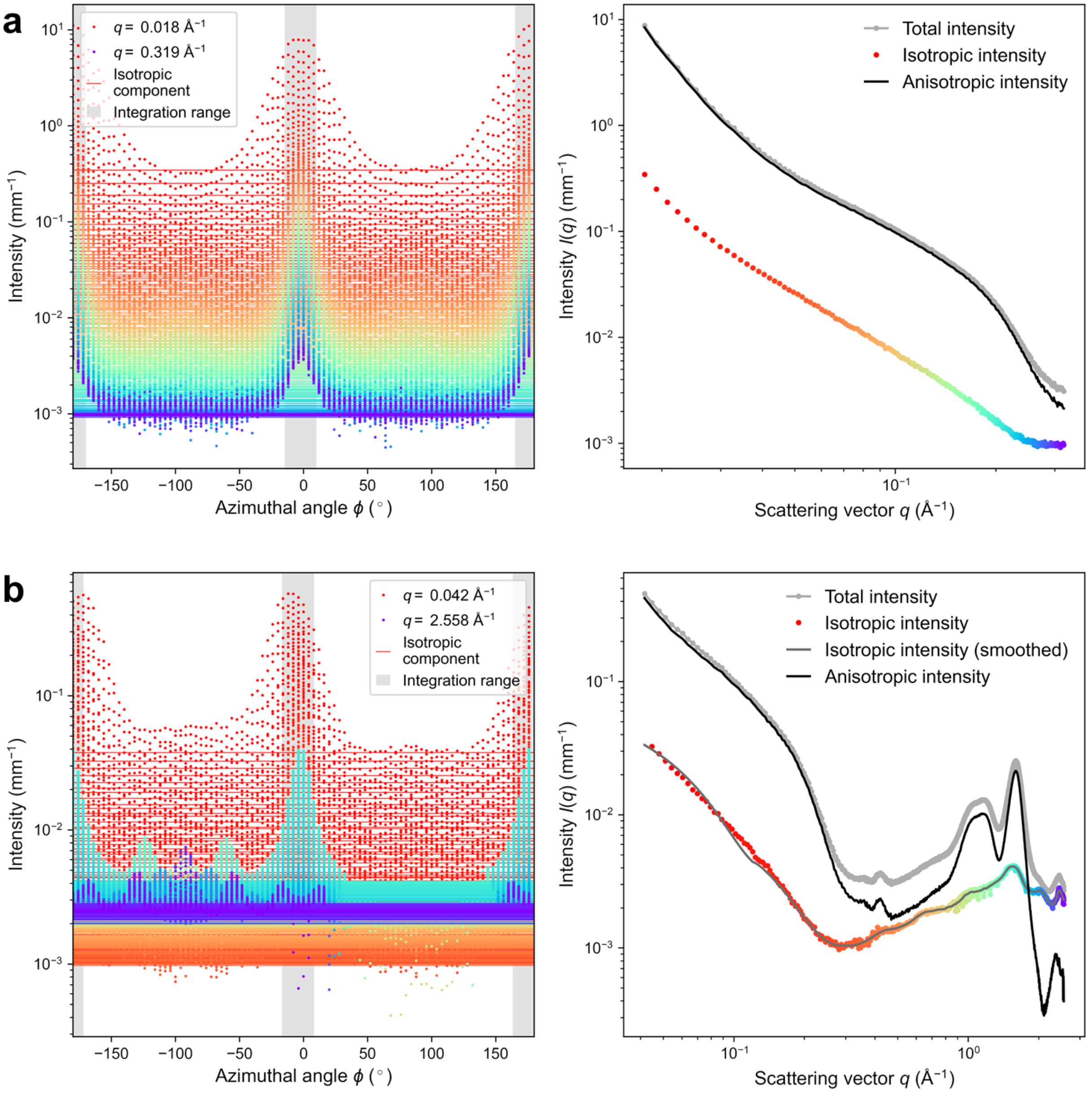

Figure S2. Separation and subtraction of isotropic scattering contribution from the equatorial SAXS (a) and WAXS (b) data, shown as an example for RH 50% (desorption).

Data analysis. For analyzing the equatorial anisotropic WAXS intensities, a linear background was subtracted between $q = 0.8$ and 2.1 Å^{-1}, and the remaining intensity was fitted with four Gaussian peaks corresponding to the 1−10, 110 and 200 (double peak) reflections of cellulose I_β (see Figure S8).[5] The double peak corresponding to the 200 reflection consisted of a broader and narrower peak with areal contributions of 70–77% and 30–22%, respectively, and including the two contributions was necessary to obtain a good fit. The double peak could be explained by a bimodal distribution of crystal size perpendicular to the plane of the glucose units ((200) plane), originating from stacking of crystals as seen in the non-periodic models (see Figure S12c,d).

The meridional anisotropic WAXS intensities were fitted with two Gaussian peaks and a constant background in the vicinity of the 004 reflection. The lattice spacing (interplanar distance) d_{hkl} corresponding to each lattice plane (*hkl*) was determined based on the peak location q_{hkl} and the Bragg law:

$$d_{hkl} = \frac{2\pi}{q_{hkl}}. \tag{S1}$$

The crystallite size L_{hkl} perpendicular to the lattice plane (*hkl*) was estimated by the Scherrer equation

$$L_{hkl} = \frac{2\pi}{\Delta q_{hkl}}, \tag{S2}$$

where Δq_{hkl} is the integral breadth of the Gaussian peak. Here Δq_{hkl} was corrected for instrumental broadening (full-width at half-maximum 0.014 Å^{-1}) as determined from a LaB_6 sample.

The equatorial anisotropic SAXS intensities were fitted using the WoodSAS model (see Figure S13):[6]

$$I(q) = A\, I_{cyl}(q, \bar{R}, \Delta R, a, \Delta a) + B\, e^{-q^2/(2\sigma^2)} + C\, q^{-\alpha} \tag{S3}$$

In Eq. S3, I_{cyl} corresponds to hexagonally packed cylinders that represent the cellulose microfibrils. The radius of the cylinders has a Gaussian distribution with mean $\bar{R}$ and standard deviation ΔR. The cylinder center points are separated by the packing distance a, which has paracrystalline distortion quantified by Δa. The second and third term of the equation correspond to larger inhomogeneities such as aggregated microfibrils or pores and surfaces of cell lumina, respectively. The parameters A, B and C are the scaling factors of the different components of the model, and the constants σ and α define the shape of the two latter contributions. The fitting was done on q-range 0.018–0.37 Å^{-1} and with fixed parameters $\Delta R/\bar{R}$=0.22 and σ=0.065 Å^{-1}.

1.4 Dynamic vapor sorption

Moisture content during scattering experiments. The moisture content (MC) of the wood sample at different RH steps of the SAXS/WAXS experiment was determined by following the same RH cycle (Figure S1) in a dynamic vapor sorption (DVS) device. A never-dried wood sample was removed from deionized water, and excess surface water was lightly wiped off the sample. The sample was then placed inside the humidity and temperature-controlled sample chamber of the DVS device (DVS ET, Surface Measurement Systems). The temperature was set to 24°C and the humidity was controlled to simulate the environment during the scattering measurements. Once this RH cycle was complete, the DVS preheater was used to heat up the sample to 60°C at 0% RH for 4.5 hours to dry it completely. This was followed by letting the sample equilibrate at 24°C and 0% RH for 60 minutes to accurately determine its dry mass. The results are reported as an average of three parallel samples with dry mass 3–6 mg.

Sorption isotherms. For the sorption isotherms of spruce wood (Figure 5 in the main article), never-dried and water-saturated wood ground with a Wiley mill was wiped to remove excess surface moisture and placed in the sample chamber of a DVS device (DVS Intrinsic, Surface Measurement Systems). Two different samples with dry masses of 5.77 mg and 20.65 mg were measured. During desorption, the samples were equilibrated for 72 h at target RH level 95%, 24 h at 85%, and 12 h at 70%, 50%, 30%, 10%, and 0%, with a 20-min moisture ramp between each level. For the adsorption, the RH was raised from 0% to 95% using the same RH steps, however, with shorter durations for each level: 6 h at 10%, 30%, 50%, and 70%, 12 h for 85%, and 24 h for 95%. The chamber temperature was 25°C for each step. The actual RH of the chamber did not always reach the exact target RH but was never off by more than 1.5 percentage points. The actual RH remained stable during the measurement, varying by less than 0.1 percentage points at any point. To determine the dry mass after the desorption–adsorption cycle was finished, the RH was brought down to 0% and the temperature was raised to 40°C with a 60 min ramp-up. The chamber was kept at 40°C for 6 hours to dry the sample. After drying, the temperature was lowered to 25°C over 60 min. The sample was then allowed to equilibrate at 0% RH for 2 h to determine the dry mass. Mass of the sample at each RH was determined by taking the average mass of the last 30 min at that RH. The actual RH was similarly the average over the last 30 min. The mass at each RH level, together with the dry mass, was then used to calculate the equilibrium MC. There were no significant differences between the two samples, aside from the very first desorption data-point at RH 95%, which was due to insufficient initial equilibration time for the sample with higher mass. The rate of change in mass ($\mathrm{d}m/\mathrm{d}t$) in a 30-min window at each RH point was on average 0.0018% min^{-1}, with the highest value of 0.006% min^{-1} at the first desorption point of the sample with higher mass.

1.5 X-ray microtomography

X-ray microtomography imaging was conducted on the same sample as used in the X-ray scattering experiments. The air-dried sample was glued to a carbon fiber rod and scanned at ambient conditions using GE Phoenix Nanotom microtomography equipment (X-ray tube voltage 60 kV and current 200 µA). A three-dimensional reconstruction with a voxel size of 1.000 µm was made from 2600 projections obtained with three times 500 ms exposure time. The portion of latewood in the sample volume studied in the SAXS/WAXS experiments was determined from binarized images as the fraction of latewood pixels from all pixels in the sample, excluding the cell lumina. Software 3D Slicer (https://www.slicer.org/) and ImageJ (https://imagej.nih.gov/ij/) were used for the analysis and visualization of the results.

1.6 Molecular simulations

Atomistic models of cellulose microfibril aggregates were used to study the effects of changing MC on spruce secondary cell wall structure (Figure S3). The models were built in three stages. In the first stage, models of cellulose microfibril segments were created. In the second stage, the microfibrils were coated with GGM and GAX chains through dynamic adsorption simulations.

Lastly, the hemicellulose-coated fibrils were arranged to form close-packed aggregates of different size. Hybrid stochastic and molecular dynamics (MD) simulations were then used to predict their response to both gradual and sudden changes in MC. The models and the simulation protocols have been previously reported by Zitting et al.,[7] and they are recapitulated below.

Models of cellulose microfibrils. The basic building block is a cellulose microfibril segment that consists of 18 cellulose chains in the hexagonal 2-3-4-4-3-2 arrangement (Figure S3a).[8] The fibrils were created algorithmically to have the internal structure of the cellulose I_β allomorph.[5] Both periodic and non-periodic fibril models were used. In the former, the cellulose chains consist of 34 glucose units, and the first unit is covalently bonded to the last across the periodic boundary of the simulation domain. In the latter, the cellulose chains have a finite length of 30 glucose units. When subjected to dynamics relaxation simulations, the non-periodic fibrils develop a right-handed twist[9] while the periodic fibrils remain in the non-twisted state (Figure S3b,c).

Models of hemicellulose-coated fibrils. Eight variants of a hemicellulose-coated fibril were created through dynamic simulation of GGM and GAX adsorption (Figure S3d,e). Four GGM and two GAX chains of 30 repeating units were distributed around a fibril segment at roughly one nanometer distance from its surface. The hemicelluloses were initially in the twofold helical screw conformation and parallel to the fibril axis, and had different side group configurations. After this, the simulation domain was filled with water and MD was simulated for 100 ns in the canonical ensemble, at 300 K temperature. The simulation leads to adsorption of the hemicellulose chains on the fibril surfaces, while maintaining the parallel alignment and the twofold helical screw conformation. The hemicellulose-cellulose mass ratio is roughly 1:4, and the GGM-GAX mass ratio roughly 2:1. Both the mass ratios and the chemical structures were chosen to represent Norway spruce hemicelluloses.[7]

Models of fibril aggregates. Two types of microfibril aggregate models were created. In the first type, periodic fibrils were arranged to form an infinite hexagonal lattice. Two variants of this type were created: one with a single microfibril (Figure S3f) and one with four microfibrils (Figure S3g). In the second type, non-periodic fibrils were arranged to form a finite close-packed aggregate. A single variant of this type was created with seven constituent fibrils (Figure S3h). In models of the periodic type, the initial microfibril packing distance was set to 5 nm, and the interfibrillar space was filled with water to create a system with an MC_c (i.e., the mass ratio of water and the carbohydrates) of roughly 100%. In the non-periodic model, the initial microfibril packing distance was set to 4.5 nm, and the fibrils were further aggregated without water in a 1 ns simulation in the canonical ensemble, at 300 K temperature. The simulation domain was then filled with water, and water sorption and swelling of the aggregate were followed in a 600 ns simulation in the isothermal-isobaric ensemble, at 300 K temperature and 1 atm external pressure. The crystallographic orientation of the cellulose crystallites around the fibril axis was initially the same in all microfibrils of a bundle, but the fibrils were free to rotate during the simulations.

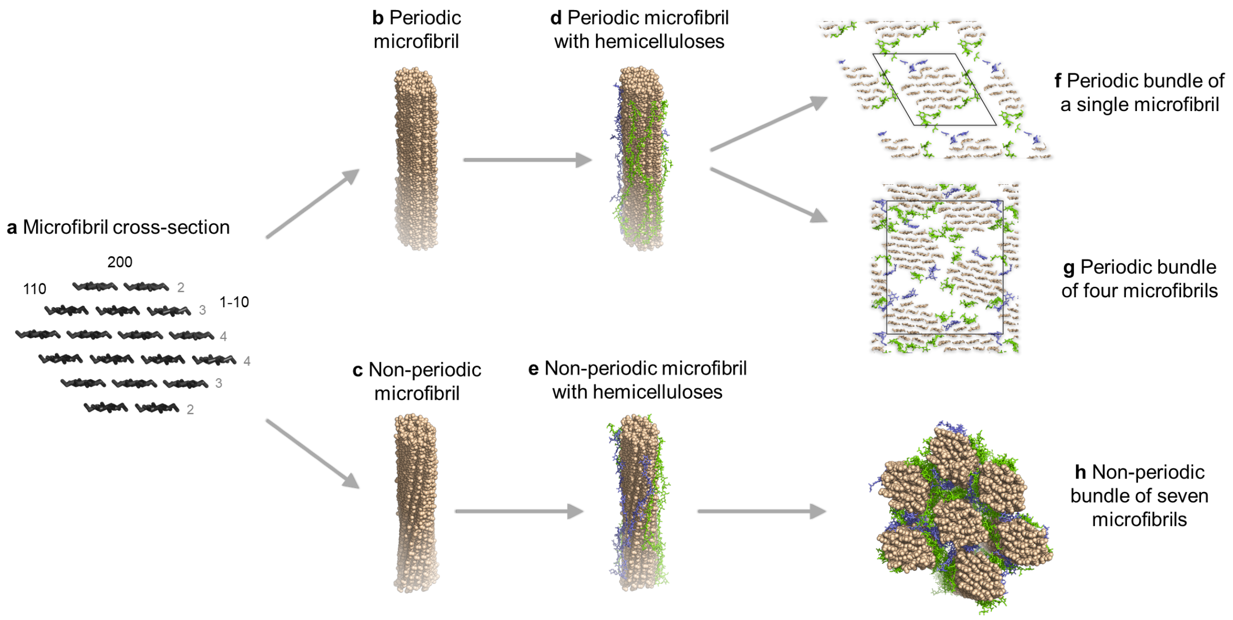

Figure S3. Molecular models of cellulose microfibrils and their aggregates. (a) Cross-section of a microfibril with cellulose chains in the 2-3-4-4-3-2 arrangement (diameter 2–3 nm). (b) Periodic and (c) non-periodic models of a microfibril segment. (d, e) Same as (b) and (c) but with adsorbed hemicelluloses (GGM in green, GAX in purple). (f) Periodic aggregate that consists of a single fibril. (g) Periodic aggregate of four fibrils. (h) Non-periodic aggregate of seven fibrils. In (f) and (g), the periodic simulation domain is outlined in black.

Simulations of drying. Hybrid stochastic-dynamic simulations were used to study the structural response of the fibril aggregates to decreasing MC_c. A drying protocol similar to that of Kulasinski et al.[10] was applied to the models of the periodic type. In the iterative drying scheme, 10 randomly chosen water molecules were removed from the system in between 100 ps simulations of MD. After each percentage point decrease in MC_c, a longer 1 ns simulation was carried out. This procedure was followed until all water was removed from the system. Sampling simulations of 600 ns were then carried out for the aggregate structures at chosen MC_cs. All simulations on systems of the periodic type were carried out in the isothermal-isobaric ensemble at 300 K temperature and 1 atm external pressure. For models of the non-periodic type, gradual drying was replaced by sudden removal of water. After this, an energy minimization was carried out. Lastly, changes in the aggregate structure were followed in a 100 ns simulation in the canonical ensemble at 300 K temperature.

Simulation set-up. The simulations were carried out using GROMACS[11] and the GLYCAM06H force field.[12] Covalent bonds involving hydrogen were constrained to a constant length using the LINCS method.[13] Water was described using the TIP3P model.[14] Temperature control was implemented using a stochastic variant of the Berendsen thermostat.[15] Pressure control was implemented using the Berendsen barostat.[16] Damping parameters of 200 fs and 2 ps were used for temperature and pressure control, respectively. The total linear momentum was reset at 2 ps

intervals. The equations of motion were integrated using the velocity-Verlet algorithm with a 2 fs time step. Molecular models of the hemicelluloses were generated using the doGlycans tool.[17]

Post-processing. Trajectory analysis was performed using GROMACS utilities, MDTraj,[18] Ovito[19] and PyMOL. Scattering intensities were calculated using in-house software. For determining the lattice spacings from the models, the molecular trajectories were coarse-grained from atomistic to sugar unit resolution. Interplanar distances were estimated at the location of each glucose unit based on its nearest-neighbor distances (Figure S4), and then averaged over the glucose units of each chain.

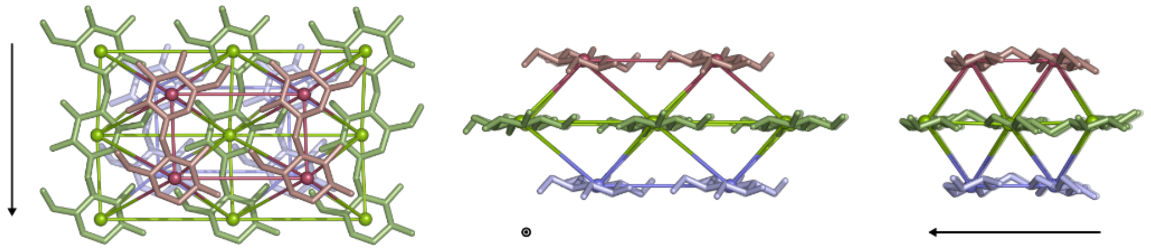

Figure S4. Lattice spacings within the fibrils were estimated based on nearest neighbor distances of the glucose units. The spheres indicate the centers of mass of the pyranose rings. The vectors adjoining them were used in different combinations to obtain local estimates for the interplanar distances d_{200}, d_{110}, d_{1-10} and d_{004}. The black arrows indicate the direction along the cellulose chains towards their reducing end (and the crystallographic *c*-axis).

1.7 Computed scattering intensities and their analysis

Scattering intensities were calculated from the models using the cylindrically symmetric form of the Debye scattering equation:[20]

$$I(q_R, q_Z) = \sum_i^n \sum_j^n f_i(q_R, q_Z) f_j(q_R, q_Z) J_0(2\pi r_{ij} q_R) \cos(2\pi z_{ij} q_Z), \qquad \text{(S4)}$$

where I is the scattering intensity, q_R and q_Z are the equatorial and meridional scattering vectors, respectively, i and j are atom indices, n is the number of atoms, f_i and f_j are atomic form factors,[21] r_{ij} and z_{ij} are the radial and axial components of interatomic distance between atoms i and j, respectively, and J_0 is the Bessel function of the first kind and order zero. Equatorial and meridional scattering intensities were calculated by setting $q_Z=0$ and $q_R=0$, respectively.

Molecular trajectories were used differently in the equatorial and meridional scattering calculation. For equatorial scattering, the fibril bundles were divided into individual microfibrils (fibril-by-fibril approach), and the microfibrils into axial segments of roughly one nanometer. The atomic coordinates of the segments were sampled at 5 ns intervals over the last 100 ns of the

sampling trajectory. Scattering intensities were calculated separately for each snapshot of each segment, and then averaged over the snapshots, segments, and the different fibrils of the bundle. The symmetry axis was chosen to coincide with the longitudinal axis of the fibril. As an exception, the scattering calculation of the non-periodic bundle of seven fibrils was carried out both with and without dividing the model into individual fibrils. In the latter case, the symmetry axis was chosen to coincide with the longitudinal axis of the bundle. For meridional scattering, the procedure was otherwise similar, but the fibrils were not divided into axial segments. The calculations were performed both with and without the adsorbed hemicelluloses. Water was not included in the analysis due to challenges arising from calculating the scattering fibril by fibril.

The purpose of the microfibril-by-microfibril and segment-by-segment calculation was to mitigate problems that arise from small system size, such as limited sampling of fibril orientations, inter-fibril distances and fibril deformations, such as bending. The fibril-by-fibril calculation allows adjusting the symmetry axis for each fibril individually, and the segment-by-segment calculation corrects for the shift of the lateral fibril cross-section due to fibril bending. Consequently, the scattering contributions from the packing of the fibrils and the fibril-fibril interfaces were omitted, but the equatorial and meridional scattering correspond better to an ideal orientation of the fibril axis in the meridional direction. This approach was chosen to make the computed scattering comparable to the experimental equatorial and meridional anisotropic WAXS intensities, which were obtained by azimuthal integration over narrow sectors around the equator and meridian, respectively, followed by subtraction of the isotropic scattering component.

Fitting of the scattering intensities computed from the models was done in a similar way as when analyzing the experimental WAXS data. A linear background was subtracted from the equatorial intensities between $q = 0.8$ and 1.9 $Å^{-1}$, and the remaining intensity was fitted with three or four Gaussian peaks corresponding to the 1−10, 110 and 200 (single or double peak) reflections. As most of the scattering intensities were computed fibril-by-fibril, only fitting the intensity computed for a complete non-periodic bundle of seven fibrils required the use of a double peak for the 200 reflection. Example fits are shown in Figure S12. The meridional intensity was fitted with one single Gaussian peak for the 004 reflection and a constant background.

2. Supplementary results

Table S1. Results of compositional analysis of the spruce wood sample, with error margins corresponding to one standard deviation

**Polysaccharides (anhydrosugars, g/100 g of sample)**							**Lignin (% of dry weight)**		**Extractives (% of dry weight)**
Glucan	Mannan	Xylan	Galactan	Arabinan	Rhamnan	Fructan	Klason lignin	Soluble lignin	
42.6 ±0.2	11.43 ±0.04	4.86 ±0.05	1.257 ±0.001	0.867 ±0.005	0.132 ±0.002	<0.1	27.0 ±0.2	0.18 ±0.02	0.69

Table S2. Evaluation of suggested mechanisms of cellulose lattice deformations with moisture changes

Material	Explanation	Relation to our results	Our conclusion
Sugi wood (*Cryptomeria japonica*)[22]	Matrix substance compresses the microfibrils transversely under green conditions (d_{200} decreases), longitudinal compressive stress induced by shrinking matrix substance (d_{004} decreases) while drying.	Nothing restricts the lateral swelling in our models, but we still observe changes in d_{200}. Our models reproduce the change of d_{004}, but the reason remains unclear.	Longitudinal change possible, lateral change unlikely
Kunugi oak (*Quercus acutissima*),[23] sugi wood (*Cryptomeria japonica*),[24] hinoki wood (*Chamaecyparis obtusa*)[24]	Lateral shrinking of matrix substances generate lateral tensile stresses to microfibrils in drying, which contribute to the increase in d_{200} lattice spacing. No change in longitudinal direction observed.	Hemicelluloses (matrix) clearly play a role in our models, but they cannot explain all the observations (e.g., distortion of crystallite surfaces by fibrillar aggregation).	Partially possible
Monterey pine (*Pinus radiata*)[25]	Dehydration of the crystal surface forces the methylol conformation to become *trans-gauche* (*tg*), so that an intrachain hydrogen bond can be formed and the packing density of chains in *b* direction increases (*b* and γ decrease). Change in *a* (d_{200}) and *c* (d_{002}) was not clear from the data.	Our models indeed show an increase of surface-chain *tg* fraction with dehydration. They also agree with the decrease in *b* with dehydration, but the changes in *a* and *c* are more consistent.	Partially possible
Norway spruce (*Picea abies*)[26]	Ordered molecular layers of water or a mixture of water and matrix components on the hydrophilic microfibril surfaces create compressive stresses (d_{200} decreases with hydration), and the compression releases with dehydration. Contraction along the *c* axis (d_{004}) could be a result of anisotropic elastic deformation of the microfibrils.	Although we acknowledge the potentially important role of order within water, our models indicate a higher level of order in the hydrated state, which suggests that the system is less stressed in wet than dry state. The 200 surface should be hydrophobic, not hydrophilic.	Partially possible
Bacterial cellulose (by *Gluconacetobacter xylinus*), cotton fibers[27]	Condensation and evaporation of ordered molecular layers of water impact the microfibril surface chains, and macroscale mechanical stresses associated with drying affect both surface and interior glucans. Change observed in direction perpendicular to hydrogen-bonded glucan sheets (d_{200} in cotton, d_{110} in bacterial cellulose), with larger change in cotton. No change in longitudinal direction detected.	Our models support the important role of interfacial water in combination with fibrillar aggregation, especially in the lateral deformations of the crystals.	Partially possible
Chlorite delignified latewood fiber enriched unbleached softwood kraft pulp[28]	Swelling forces, which are created in the hydrated state by combined longitudinal and lateral expansion of the non-crystalline cellulose molecules and aligned hemicelluloses, cause lateral contraction (d_{200} decreases) and longitudinal extension (d_{004} increases).	Nothing restricts the lateral swelling in our models, but we still observe changes in d_{200}. Our model fibrils had better crystalline order in the hydrated state.	Unlikely

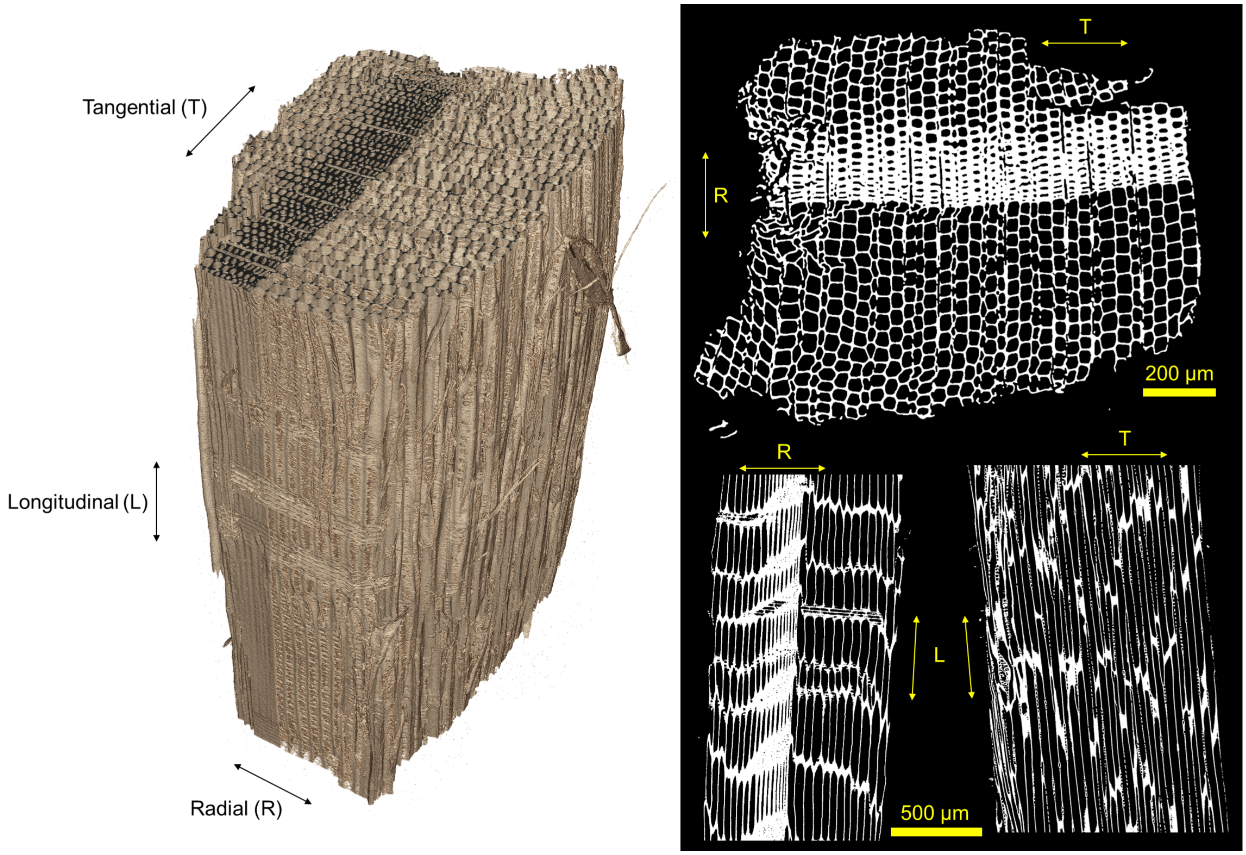

Figure S5. Three-dimensional reconstruction of the X-ray microtomography data of the wood sample used in the SAXS/WAXS experiments (left) along with three orthogonal slices (right).

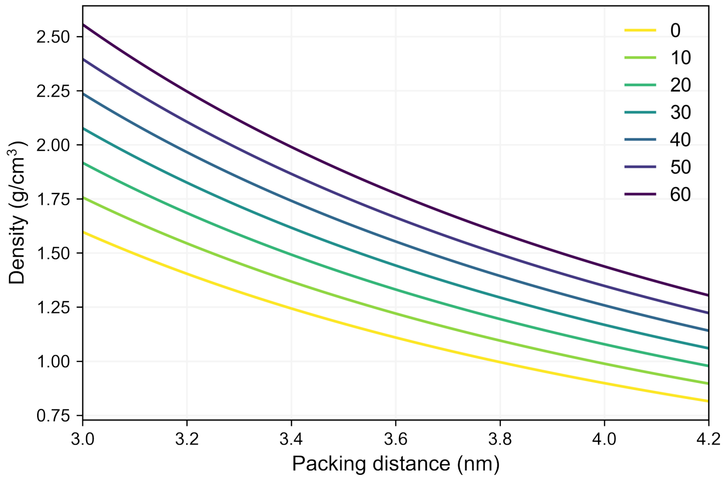

Figure S6. Estimates for the density of a microfibril bundle at different MCs (in %) and microfibril packing distances. The calculation assumes 18 cellulose chains in a fibril, hemicellulose content 25 wt%, hexagonal packing of the fibrils, and densities of 1.62, 1.5 and 1.0 g/cm^3 for cellulose, hemicelluloses and water, respectively.

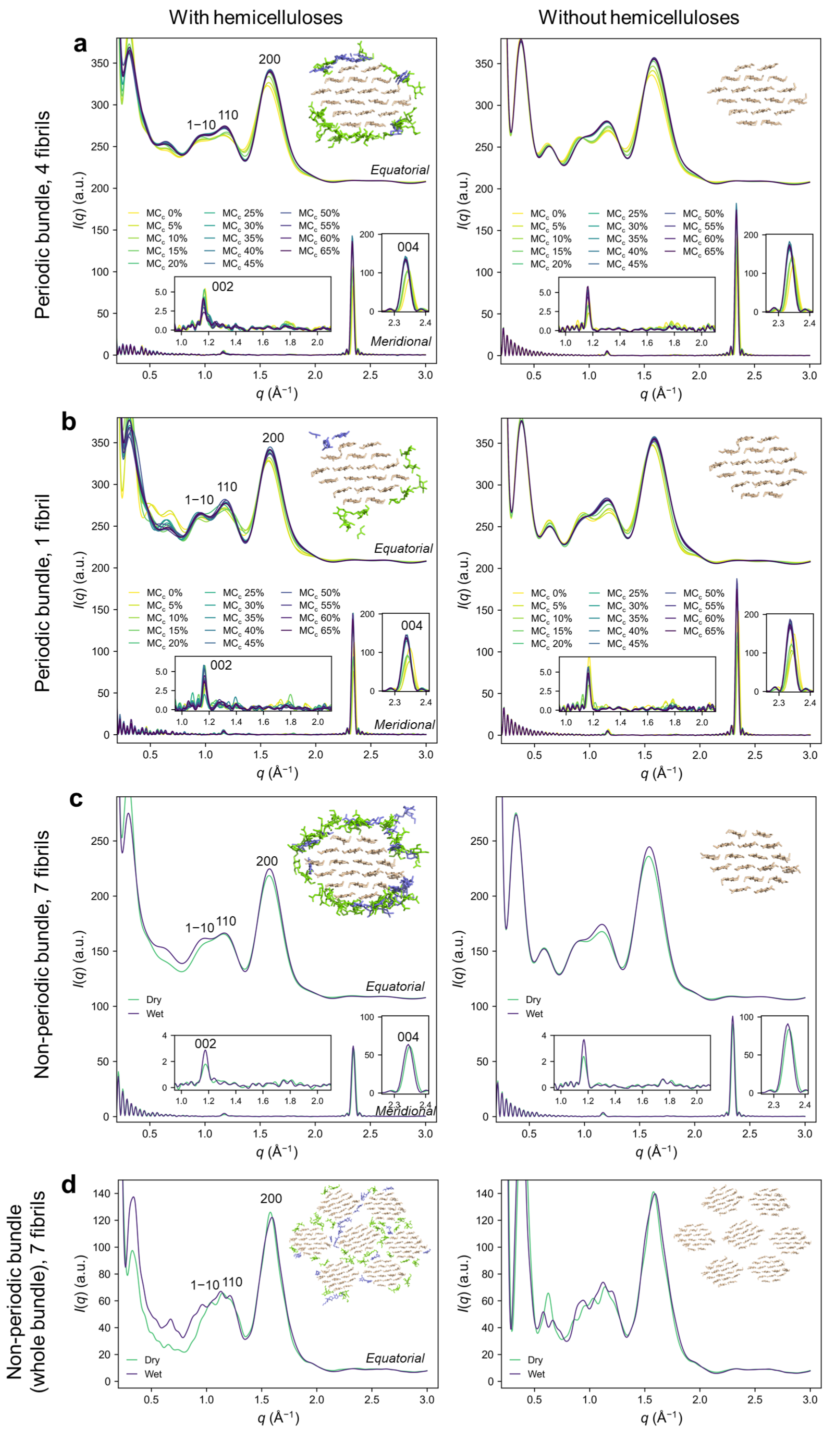

Figure S7. Computed scattering intensities based on the MD models, including (left) or excluding (right) the contribution from hemicelluloses. (a) Periodic bundle model of four fibrils, with scattering computed fibril-by-fibril. (b) Periodic bundle model of one fibril. (c) Non-periodic bundle model of seven fibrils, with scattering computed fibril-by-fibril. (d) Model of (c), with scattering computed from the whole bundle. In (a) and (c), the insets show a superposition of the fibrils' hemicellulose configurations.

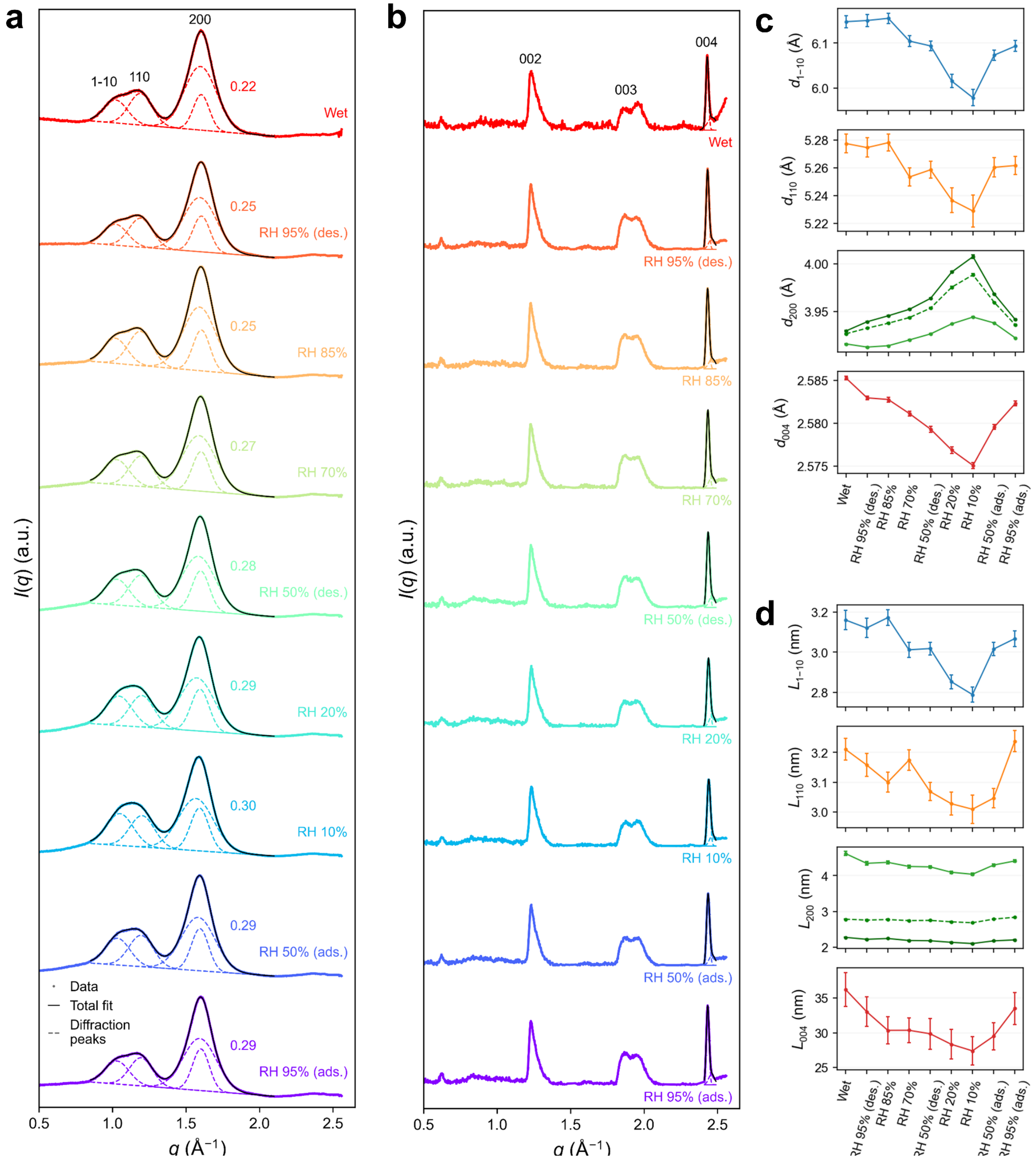

Figure S8. Results of fitting to the experimental WAXS data. (a, b) Fits to the equatorial (a) and meridional (b) anisotropic WAXS intensities. (c) Lattice spacings (d_{hkl}) and (d) crystal size (L_{hkl}) determined from the WAXS intensities by peak fitting. The decimal number next to the 200 peak in (a) marks the areal fraction of the narrower peak in the total 200 peak. In (c) and (d), results for the 200 reflection are shown separately for the narrow (light green) and broad peak (dark green) and their weighted mean (green with dashed line).

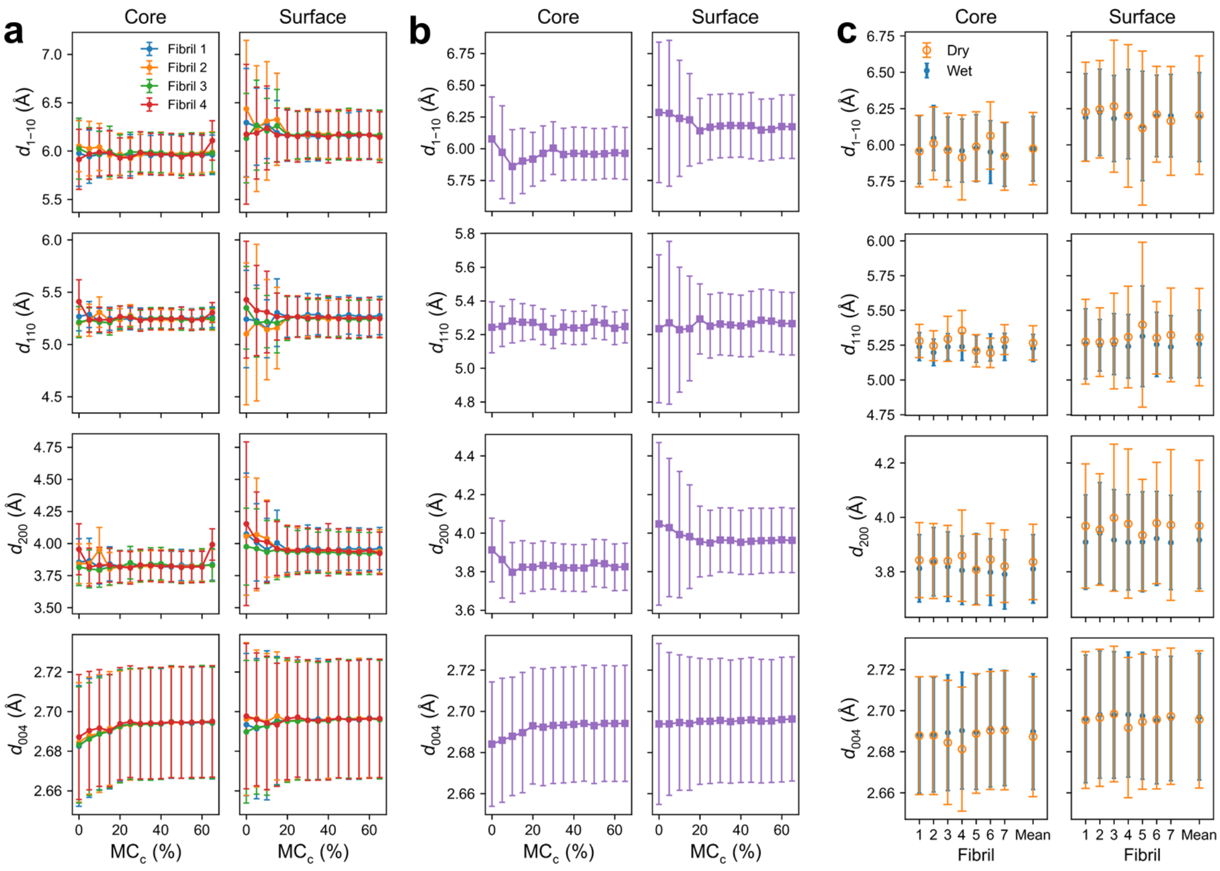

Figure S9. Lattice spacings d_{hkl} determined directly from the models, shown separately for core and surface chains. (a) Periodic bundle model of four fibrils. (b) Periodic bundle model of a single fibril. (c) Non-periodic model of seven fibrils in wet and dry states. Mean lattice spacing (symbols) and its standard deviation (error bars) are presented for each fibril, except for the "Mean" in (c), which shows the mean of all seven fibrils and their standard deviations.

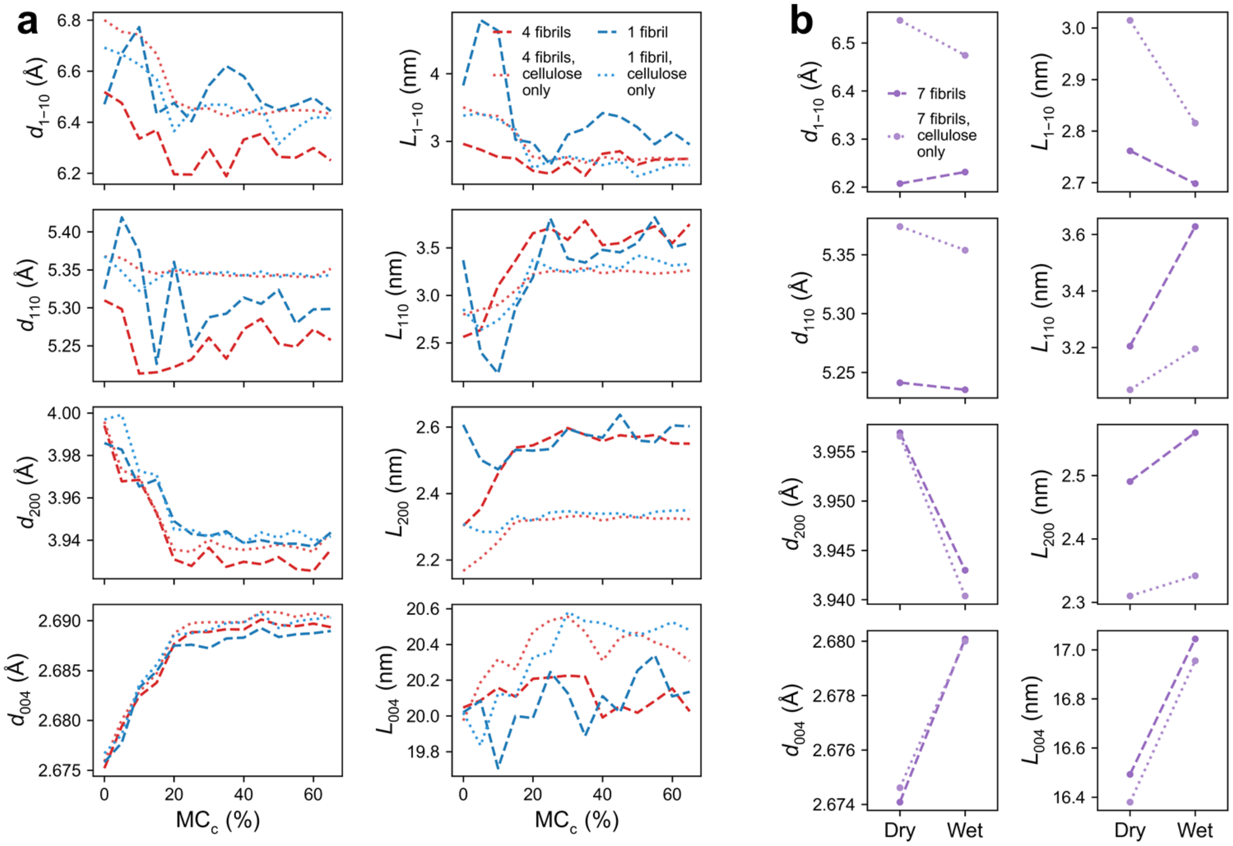

Figure S10. Lattice spacings (d_{hkl}) and crystal size (L_{hkl}) determined from the computed scattering intensities by peak fitting, either with or without the scattering contribution of hemicelluloses. (a) Periodic bundle models of one and four fibrils. (b) Non-periodic bundle model of seven fibrils.

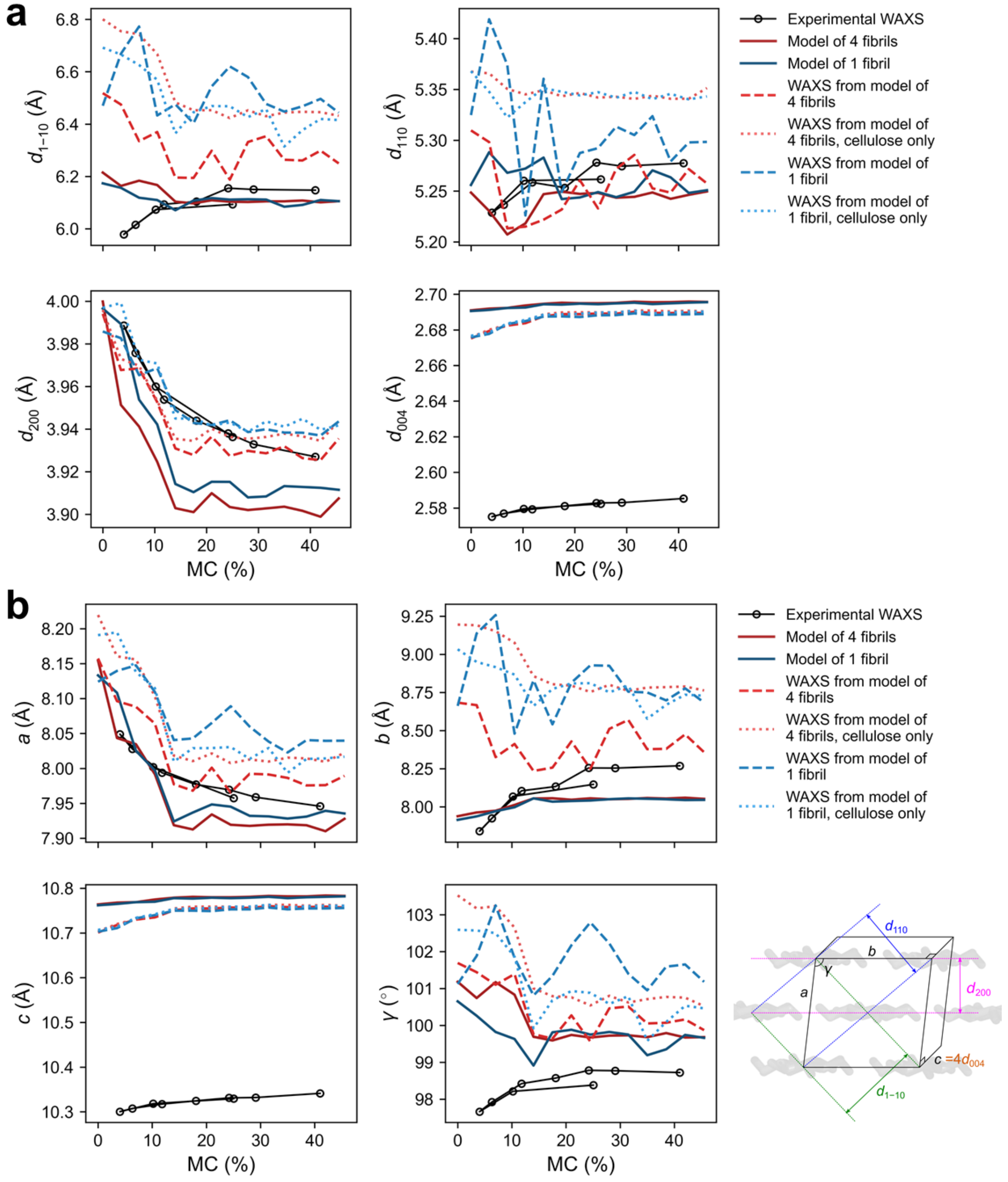

Figure S11. Comparison between crystalline parameters determined experimentally and from the models. (a) Lattice spacings determined experimentally and from the periodic bundle models either directly or through fits to computed scattering data (the experimental result for d_{200} corresponds to the weighted mean of the two fitted peaks). (b) Lattice parameters of a monoclinic unit cell calculated from the lattice spacings in (a) according to the geometry shown on the lower right. The MC for the models (MC_c) has been multiplied by 0.7 to roughly compare it with experimental data from complete cell walls (30 wt% non-water sorbing material).

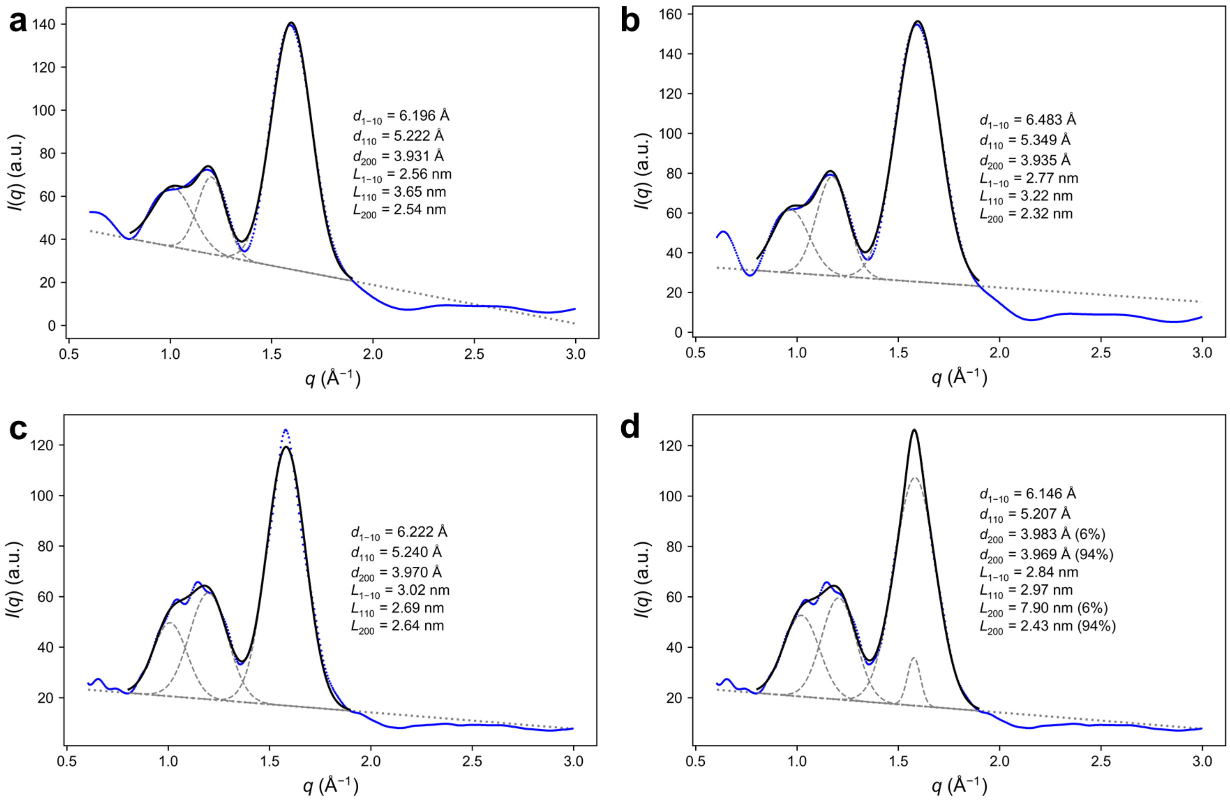

Figure S12. Example peak fits to computed equatorial scattering intensities. (a) Periodic bundle model of four fibrils including hemicelluloses at MC_c 20%. (b) Same as in (a) but excluding hemicelluloses. (c) Non-periodic bundle of seven fibrils including hemicelluloses (dry), fitted with one peak for the 200 reflection. (d) Same as in (c) but fitted with two peaks for the 200 reflection.

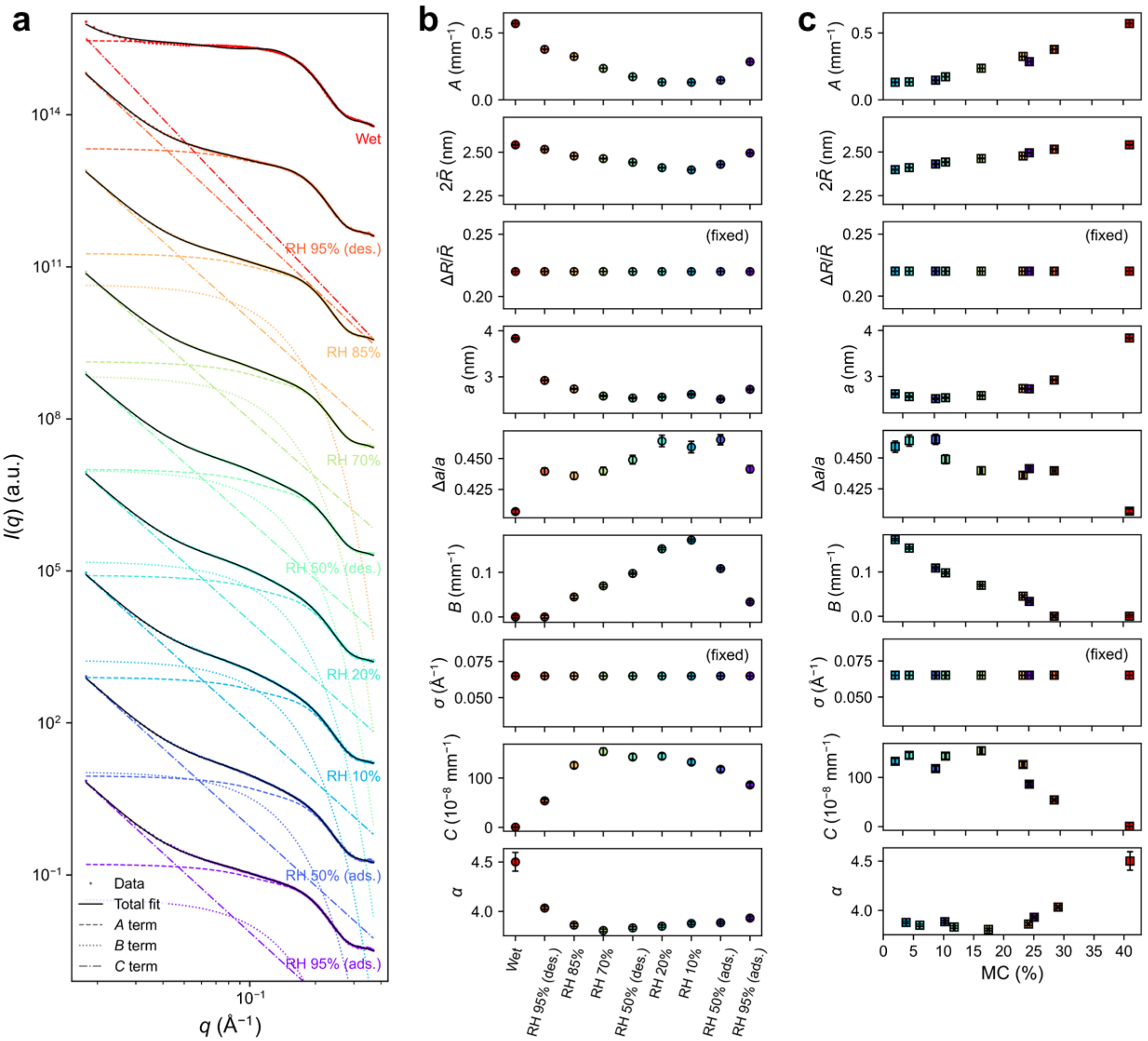

Figure S13. Results of fitting to the equatorial anisotropic SAXS intensities. (a) Total fits and the contributions of different terms of the WoodSAS model (Eq. S3). (b, c) The resulting fitting parameters at different moisture conditions (b) and as a function of MC (c).

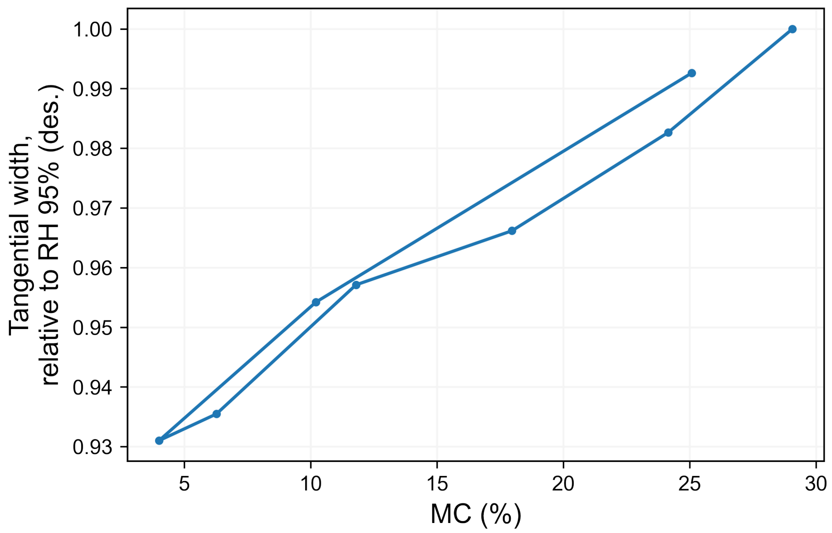

Figure S14. Change in the macroscopic width of the wood sample as determined by horizontal scanning with the X-ray beam. A linear fit yielded a slope of 0.27 percentage points per MC percentage point.

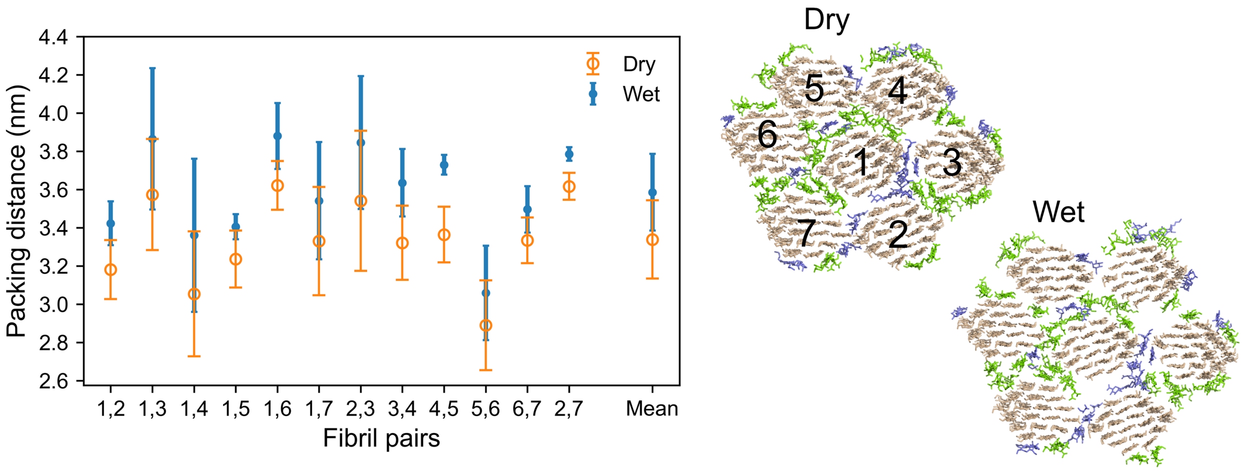

Figure S15. Microfibril packing distance (left) as determined for fibril pairs in the non-periodic bundle model of seven fibrils (right), with the error bars presenting the standard deviation.

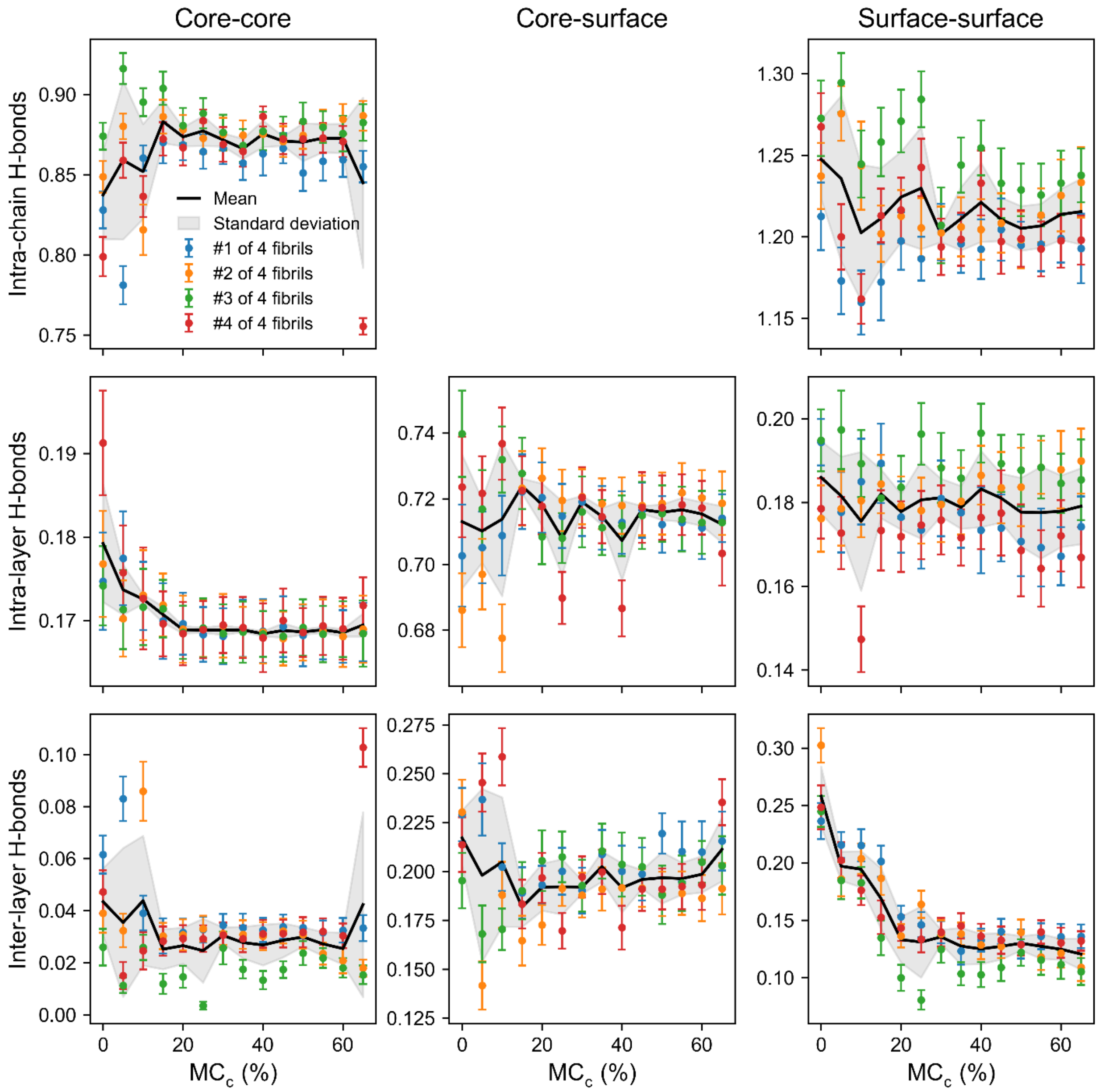

Figure S16. Number of intra-chain (top row), intra-layer (middle row) and inter-layer (bottom row) hydrogen bonds per glucose between core chains (left column), core and surface chains (middle column), and surface chains (right column) as a function of MC$_c$, as determined from the periodic model of four fibrils. Intra-layer bonds do not include intra-chain bonds within the layers. The most prominent effect of drying is the increase of inter-layer hydrogen bonds within the surface chains. This implies deformation of the fibril surfaces, such as shown in Figure 4e of the main article. Changes in intra-chain hydrogen bonds reflect changes in the *trans-gauche* (*tg*) conformer fraction of the primary alcohol groups, as shown in Figure 4d and Figure S17.

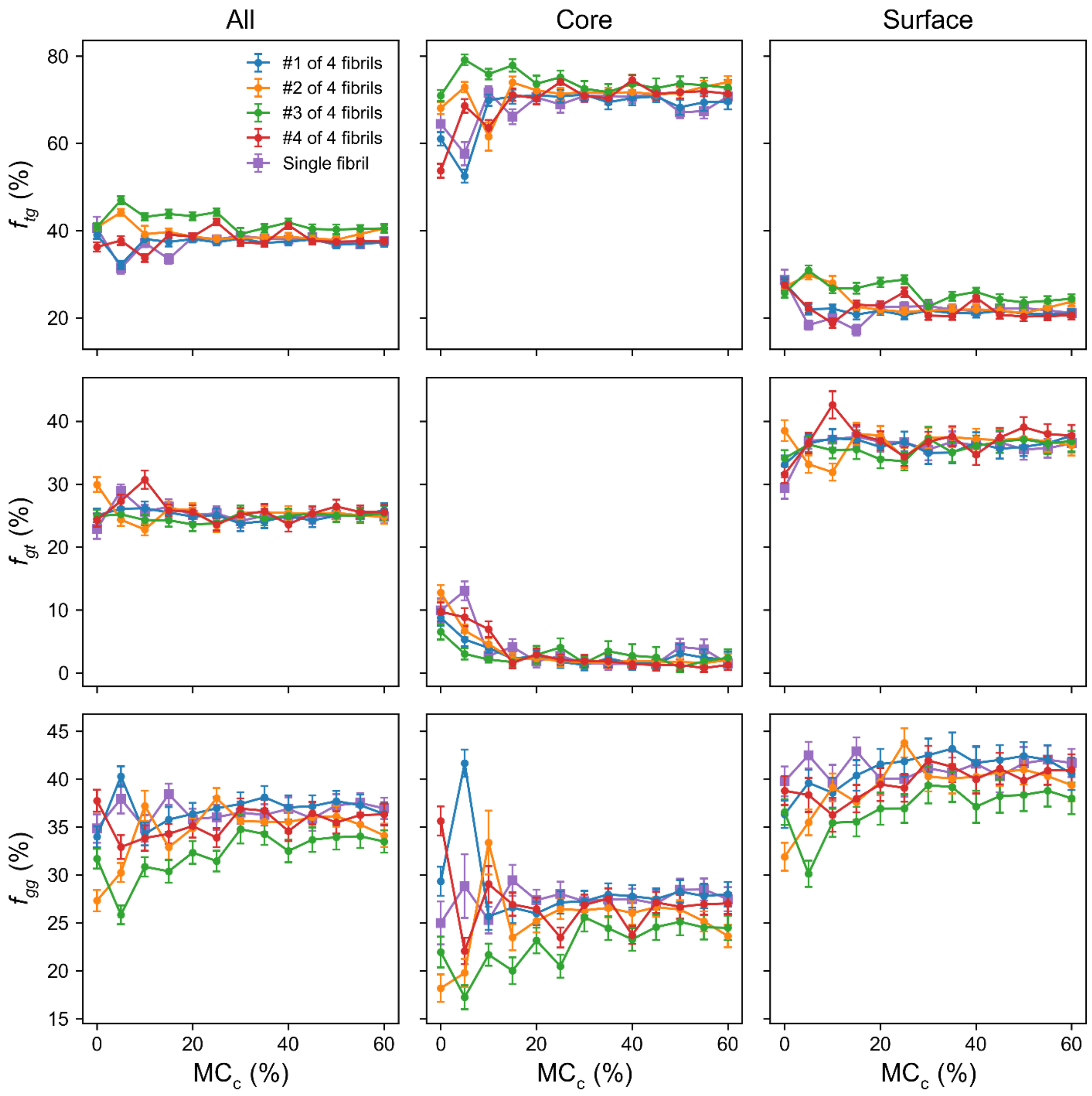

Figure S17. Fractions (*f* in %) of primary alcohol group conformers (*tg*, *trans-gauche*; *gt*, *gauche-trans*; *gg*, *gauche-gauche*) as a function of MC$_c$, as determined from the four-fibril and single-fibril periodic models for all cellulose chains (left) and separately for core (center) and surface chains (right). Conformational disorder occurs throughout the fibrils, but it is most prominent at the fibril surfaces, as indicated by the low *tg* fractions. The effects of drying include increased disorder in the core chains.

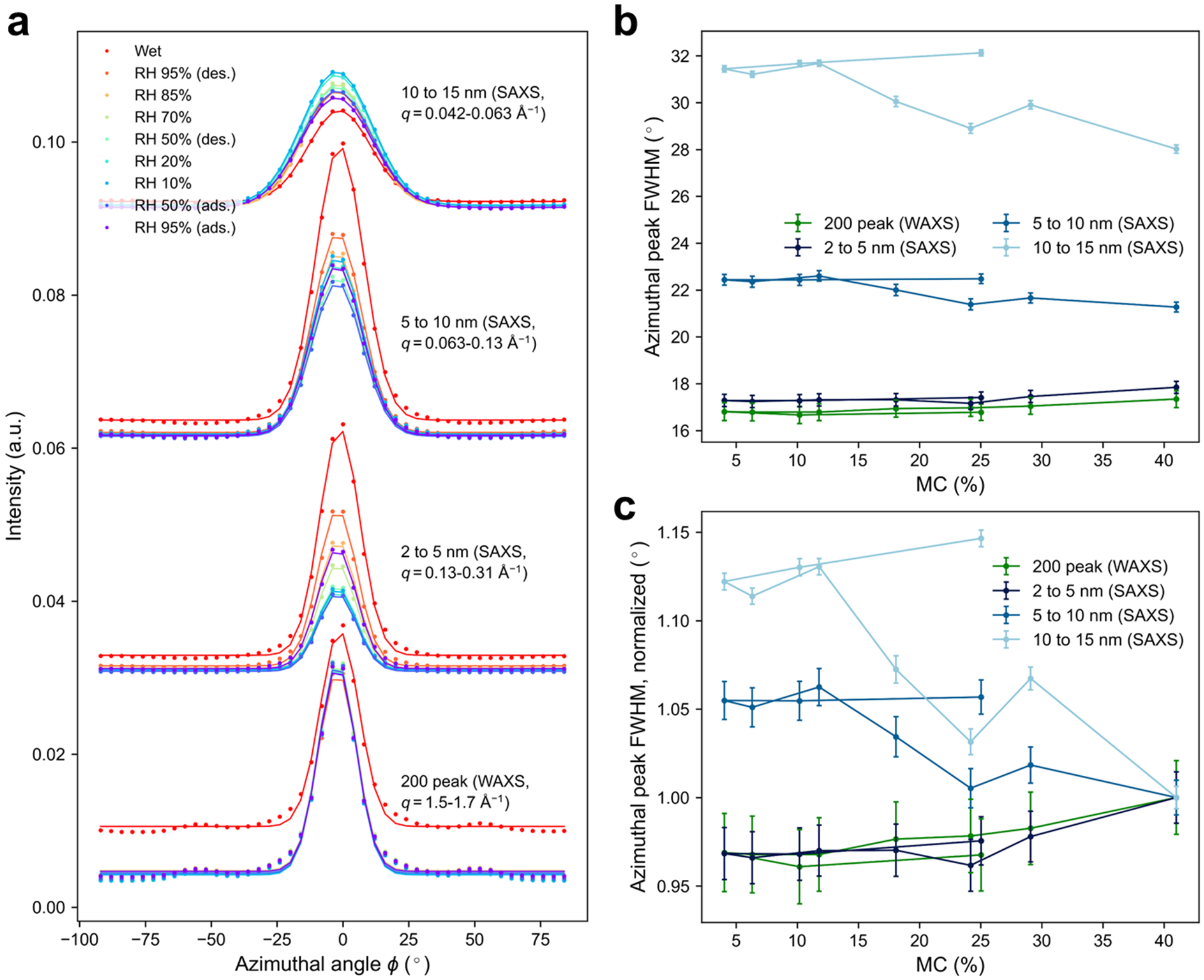

Figure S18. Analysis of azimuthal intensity profiles from SAXS and WAXS data to characterize the orientational distribution of the microfibrils. (a) Azimuthal intensity profiles (points), radially integrated over q-ranges corresponding to the 200 diffraction peak and to structures of different length scales (arbitrarily scaled and shifted for visualization) and fits of Gaussian functions (solid lines). (b) Full-width at half-maximum (FWHM) of azimuthal peaks (based on the Gaussian fits) as a function of MC. (c) Same as (b) but normalized to the value in fully wet state. The individual microfibrils (2–5 nm) and cellulose crystallites (200 peak) were slightly more uniformly oriented at low MCs (3% change from wet to lowest MC). The changes in the azimuthal intensities at lower q-values (5–10 nm and 10–15 nm) showed opposite trends, which may reflect changes in the matrix structure.

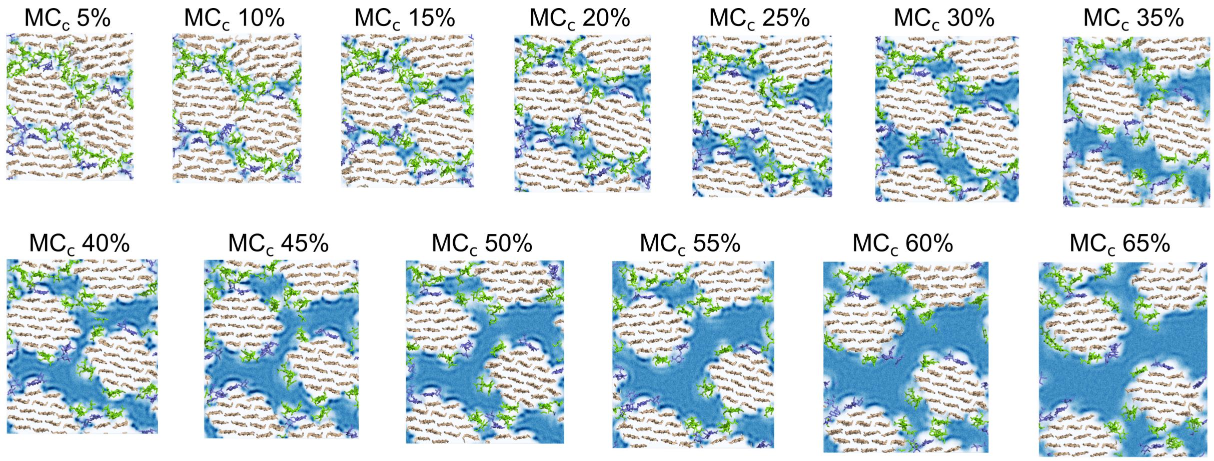

Figure S19. Simulation snapshots of the periodic bundle model of four fibrils, illustrating the changes with moisture.

References

1. Sluiter, A.; Hames, B.; Ruiz, R.; Scarlata, C.; Sluiter, J.; Templeton, D.; Crocker, D. *Determination of Structural Carbohydrates and Lignin in Biomass, Laboratory analytical procedure (LAP)*; National Renewable Energy Laboratory (NREL), 2008.
2. Hausalo, T. (1995). "Analysis of wood and pulp carbohydrates by anion exchange chromatography with pulsed amperometric detection", Proceedings of the 8th ISWPC, June 6-9, 1995, Helsinki, Finland, pp. 131-136.
3. Goldschmid, O. Ultraviolet Spectra. In *Lignins: Occurrence, Formation, Structure and Reactions*; Sarkanen, K.V., Ludwig, C.H., Eds. John Wiley & Sons, New York, 1971. Chap. 6, p. 241-298.
4. Kieffer, J.; Valls, V.; Blanc, N.; Hennig, C. New tools for calibrating diffraction setups. J. Synchrotron Radiat. 2020, 27, 558–566.
5. Nishiyama, Y.; Langan, P.; Chanzy, H. Crystal structure and hydrogen-bonding system in cellulose Iβ from synchrotron X-ray and neutron fiber diffraction. J. Am. Chem. Soc. 2002, 124, 9074–9082.
6. Penttilä, P. A.; Rautkari, L.; Österberg, M.; Schweins, R. Small-angle scattering model for efficient characterization of wood nanostructure and moisture behaviour. J. Appl. Crystallogr. 2019, 52, 369–377.
7. Zitting, A.; Paajanen, A.; Rautkari, L.; Penttilä, P. A. Deswelling of microfibril bundles in drying wood studied by small-angle neutron scattering and molecular dynamics. Cellulose 2021, 28, 10765–10776.
8. Yang, H.; Kubicki, J. D. A density functional theory study on the shape of the primary cellulose microfibril in plants: effects of C6 exocyclic group conformation and H-bonding. Cellulose 2020, 27, 2389–2402.
9. Matthews, J. F.; Skopec, C. E.; Mason, P. E.; Zuccato, P.; Torget, R. W.; Sugiyama, J.; Himmel, M. E.; Brady, J. W. Computer simulation studies of microcrystalline cellulose Iβ. Carbohydr. Res. 2006, 341, 138–152.
10. Kulasinski, K.; Guyer, R.; Derome, D.; Carmeliet, J. Water adsorption in wood microfibril-hemicellulose system: role of the crystalline–amorphous interface. Biomacromolecules 2015, 16, 2972–2978.
11. Abraham, M. J.; Murtola, T.; Schulz, R.; Páll, S.; Smith, J. C.; Hess, B.; Lindahl, E. GROMACS: High performance molecular simulations through multi-level parallelism from laptops to supercomputers. SoftwareX 2015, 1–2, 19–25.
12. Kirschner, K. N.; Yongye, A. B.; Tschampel, S. M.; González-Outeiriño, J.; Daniels, C. R.; Foley, B. L.; Woods, R. J. GLYCAM06: A generalizable biomolecular force field. Carbohydrates. J. Comput. Chem. 2008, 29, 622–655.
13. Hess, B.; Bekker, H.; Berendsen, H. J. C.; Fraaije, J. G. E. M. LINCS: A linear constraint solver for molecular simulations. J. Comput. Chem. 1997, 18, 1463–1472.
14. Jorgensen, W. L.; Chandrasekhar, J.; Madura, J. D.; Impey, R. W.; Klein, M. L. Comparison of simple potential functions for simulating liquid water. J. Chem. Phys. 1983, 79, 926–935.

15. Bussi, G.; Donadio, D.; Parrinello, M. Canonical sampling through velocity rescaling. J. Chem. Phys. 2007, 126, 014101.

16. Berendsen, H. J. C.; Postma, J. P. M.; van Gunsteren, W. F.; DiNola, A.; Haak, J. R. Molecular dynamics with coupling to an external bath. J. Chem. Phys. 1984, 81, 3684–3690.

17. Danne, R.; Poojari, C.; Martinez-Seara, H.; Rissanen, S.; Lolicato, F.; Róg, T., Vattulainen, I. doGlycans–Tools for preparing carbohydrate structures for atomistic simulations of glycoproteins, glycolipids, and carbohydrate polymers for GROMACS. J. Chem. Inf. Model. 2017, 57, 2401–2406.

18. McGibbon, R. T.; Beauchamp, K. A.; Harrigan, M. P.; Klein, C.; Swails, J. M.; Hernández, C. X.; Schwantes, C. R.; Wang, L.-P.; Lane, T. J.; Pande, V. S. MDTraj: A modern open library for the analysis of molecular dynamics trajectories. Biophys. J., 2015, 109, 1528–1532.

19. Stukowski, A. Visualization and analysis of atomistic simulation data with OVITO–the Open Visualization Tool. Modell. Simul. Mater. Sci. Eng. 2009, 18, 015012.

20. Zhang, Y.; Inouye, H.; Crowley, M.; Yu, L.; Kaeli, D.; Makowski, L. Diffraction pattern simulation of cellulose fibrils using distributed and quantized pair distances. J. Appl. Crystallogr. 2016, 49, 2244–2248.

21. International Tables for Crystallography. Volume C: Mathematical, Physical and Chemical Tables. Kluwer Academic Publishers, Dordrecht/Boston/London 1992 (published for the International Union of Crystallography).

22. Abe, K.; Yamamoto, H. Change in mechanical interaction between cellulose microfibril and matrix substance in wood cell wall induced by hygrothermal treatment. J. Wood Sci. 2006, 52, 107–110.

23. Yamamoto, H.; Ruelle, J.; Arakawa, Y.; Yoshida, M.; Clair, B.; Gril, J. Origin of the characteristic hygro-mechanical properties of the gelatinous layer in tension wood from Kunugi oak (*Quercus acutissima*). Wood Sci. Technol. 2010, 44, 149–163.

24. Toba, K.; Yamamoto, H.; Yoshida, M. Mechanical interaction between cellulose microfibrils and matrix substances in wood cell walls induced by repeated wet-and-dry treatment. Cellulose 2012, 19, 1405–1412.

25. Hill, S. J.; Kirby, N. M.; Mudie, S. T.; Hawley, A. M.; Ingham, B.; Franich, R. A.; Newman, R. H. Effect of drying and rewetting of wood on cellulose molecular packing. Holzforschung 2010, 64, 421–427.

26. Zabler, S.; Paris, O.; Burgert, I.; Fratzl, P. Moisture changes in the plant cell wall force cellulose crystallites to deform. J. Struct. Biol. 2010, 171, 133–141.

27. Fang, L.; Catchmark, J. M. Structure characterization of native cellulose during dehydration and rehydration. Cellulose 2014, 21, 3951–3963.

28. Salmén, L.; Stevanic, J. S.; Holmqvist, C.; Yu, S. Moisture induced straining of the cellulosic microfibril. Cellulose 2021, 28, 3347–3357.
